# Medical imaging for plantar heel pain: a systematic review and meta-analysis

**DOI:** 10.1186/s13047-021-00507-2

**Published:** 2022-01-22

**Authors:** Chris Drake, Glen A. Whittaker, Michelle R. Kaminski, John Chen, Anne-Maree Keenan, Michael S. Rathleff, Philip Robinson, Karl B. Landorf

**Affiliations:** 1 Physiotherapy Department, Mid-Yorkshire Hospitals NHS Trust, Wakefield, England; 2 Discipline of Podiatry, School of Allied Health, Human Services and Sport, La Trobe University, Victoria, 3086 Australia; 3 La Trobe Sport and Exercise Medicine Research Centre, La Trobe University, Victoria, 3086 Australia; 4 Department of Rehabilitation, National University Hospital, 5 Lower Kent Ridge Road, Singapore, 119074 Singapore; 5 NIHR Leeds Biomedical Research Centre, Leeds Teaching Hospitals Trust, School of Healthcare, University of Leeds, Leeds, England; 6 Center for General Practice at Aalborg University, Fyrkildevej 7, 9220 Aalborg East, Denmark; 7 Department of Occupational Therapy and Physiotherapy, Aalborg University Hospital, Hobrovej 18-22, 9100 Aalborg, Denmark; 8 Department of Health Science and Technology, Faculty of Medicine, Aalborg University, Aalborg, Denmark

**Keywords:** Feet, Plantar heel pain, Plantar fasciitis, Medical imaging, X-rays, Scintigraphy, Ultrasound, Sonoelastography, MRI scans

## Abstract

**Background:**

Medical imaging can be used to assist with the diagnosis of plantar heel pain. The aim of this study was to synthesise medical imaging features associated with plantar heel pain.

**Methods:**

This systematic review and meta-analysis conducted searches in MEDLINE, CINAHL, SPORTDiscus, Embase and the Cochrane Library from inception to 12th February 2021. Peer-reviewed articles of cross-sectional observational studies written in English that compared medical imaging findings in adult participants with plantar heel pain to control participants without plantar heel pain were included. Study quality and risk of bias was assessed using the National Institutes of Health quality assessment tool for observational cohort and cross-sectional studies. Sensitivity analyses were conducted where appropriate to account for studies that used unblinded assessors.

**Results:**

Forty-two studies (2928 participants) were identified and included in analyses. Only 21% of studies were rated ‘good’ on quality assessment. Imaging features associated with plantar heel pain included a thickened plantar fascia (on ultrasound and MRI), abnormalities of the plantar fascia (on ultrasound and MRI), abnormalities of adjacent tissue such as a thickened loaded plantar heel fat pad (on ultrasound), and a plantar calcaneal spur (on x-ray). In addition, there is some evidence from more than one study that there is increased hyperaemia within the fascia (on power Doppler ultrasound) and abnormalities of bone in the calcaneus (increased uptake on technetium-99 m bone scan and bone marrow oedema on MRI).

**Conclusions:**

People with plantar heel pain are more likely to have a thickened plantar fascia, abnormal plantar fascia tissue, a thicker loaded plantar heel fat pad, and a plantar calcaneal spur. In addition, there is some evidence of hyperaemia within the plantar fascia and abnormalities of the calcaneus. Whilst these medical imaging features may aid with diagnosis, additional high-quality studies investigating medical imaging findings for some of these imaging features would be worthwhile to improve the precision of these findings and determine their clinical relevance.

**Supplementary Information:**

The online version contains supplementary material available at 10.1186/s13047-021-00507-2.

## Background

Plantar heel pain (PHP) is a term used to describe a prevalent, painful condition localised to the plantar aspect of the heel, which is exacerbated by weightbearing [1]. A recent study estimated the prevalence of PHP was 9.6% of the population aged 50 years or older, with 7.9% of the population reporting symptoms classified as disabling [2]. PHP is known to negatively impact health-related quality of life and limit activity levels [3]. It has also been found to have a substantial financial and health service burden [4–7].

Various risk factors for PHP have been described, although only body mass index (BMI) appears to be consistently associated with symptoms [8, 9]. Often thought to be a short to medium term self-limiting condition, one study recently documented that symptoms can last much longer than originally thought – up to 10 years for half of the participants [10]. Because patients are often uncertain about the cause and prognosis of PHP, they can feel confused about their symptoms and have unmet needs and expectations regarding their care [11]. In particular, early in the patient journey, the diagnosis of the condition and cause of the symptoms can be somewhat confusing for patients. Diagnosis of PHP is generally based on the clinical presentation and often targeted with the multimodal management approach [12, 13]. However, imaging can aid the identification of the tissues involved, which has the potential to target management more effectively.

Our previous systematic review of medical imaging features of PHP – now over a decade old – described several imaging features that are associated with PHP on plain film x-ray, ultrasound, MRI and scintigraphy [14]. Since this review, however, there have been advances in medical imaging, including new modalities, and a substantial number of additional imaging studies of PHP have been published across all imaging modalities. Accordingly, an updated review of multi-modality medical imaging features of PHP would improve our understanding of the condition, which may aid in identifying imaging-based subsets of the condition. Such subsets could potentially provide targets for a more personalised approach to treatment.

The aim of this systematic review was to synthesise medical imaging features associated with PHP.

## Methods

### Registration

The protocol of this systematic review was prospectively registered with PROSPERO (Registration No. CRD42020172398) and has been reported in accordance with the Preferred Reporting Items for Systematic Reviews and Meta-analyses (PRISMA) guidelines [15].

### Search strategy

Searches were conducted in MEDLINE, CINAHL, SPORTDiscus, Embase and the Cochrane Library from inception to 12th February 2021 – Additional file 1. Citation tracking using Google Scholar was performed to identify any further relevant citations. Reference lists were screened for studies not identified in the initial search.

### Eligibility criteria

Eligible articles were peer-reviewed studies published in the English language. Studies had to be cross-sectional observational studies that compared medical imaging findings from a group of adult participants with PHP to an independent control group of adult participants without PHP.

Studies were excluded if they exclusively compared a symptomatic foot with the contralateral asymptomatic foot of the same participant (e.g. no independent control group comparison) – this was done to avoid confounding where the condition may have been developing in the contralateral foot but was still asymptomatic. Studies were also excluded if they included participants who had any self-reported inflammatory arthritis (e.g. seronegative arthropathy), endocrine/neurological condition (e.g. diabetic peripheral neuropathy), surgery (e.g. joint fusion), or trauma (e.g. major fractures) that had affected lower limb sensation or their ability to walk/run and if relevant to the imaging modality of interest. The same exclusion criteria were applied to the control group without PHP in each study, who were also required to be asymptomatic of PHP on both feet.

### Study selection

The search results were exported from the bibliographic databases into Endnote X9 (Thomson Reuters, New York, USA) and duplicate citations were removed. Two authors (CD and JC) examined all the study titles and abstracts independently, and studies deemed ineligible were excluded. The full text articles of the remaining studies were obtained and examined against the eligibility criteria for inclusion in the systematic review. If consensus agreement could not be agreed between the two authors, a third author (KL) was consulted to resolve the disagreement.

### Data extraction

A data extraction form was implemented to extract the individual study characteristics (e.g. BMI) and the imaging modality (e.g. ultrasound). The primary variables of interest included: plantar fascia thickness on ultrasound and MRI, hypoechogenicity on ultrasound, plantar fascia tear on ultrasound and MRI, plantar fascia stiffness on sonoelastography, hyperintensity on MRI, hyperaemia on power Doppler ultrasound, plantar intrinsic muscle size on ultrasound and MRI, plantar calcaneal spur on x-ray, bone marrow oedema on MRI, calcaneal crescent sign on MRI, and radioisotope uptake on scintigraphy. Variables that could have led to bias were also extracted (e.g. blinding). Two authors (CD and GW) independently extracted and compared their data to minimise errors. A third author (KL) was consulted when consensus on the data extracted could not be reached.

### Quality appraisal

The National Institutes of Health (NIH) quality assessment tool for observational cohort and cross-sectional studies was used to assess study quality and risk of bias [16]. The tool has 14 questions that are specific to cross-sectional studies (the study type included in our review), which encompass the key concepts required to investigate the internal validity of a study (selection, information, measurement and confounding bias). The tool allows a rating to be applied to a study (rated as ‘poor’, ‘fair’ or ‘good’) based on individual details and consideration of the concepts, rather than a tally scoring system. Low risk of bias equates to a ‘good’ quality rating, whereas high risk of bias equates to a ‘poor’ quality rating. Two authors (CD and MK) independently performed the quality assessment and disagreements were resolved through consensus. A third author (KL) was to be consulted when consensus could not be reached, however this was not required.

### Data analysis

Meta-analyses were performed using Review Manager (RevMan, Version 5.4, The Cochrane Collaboration, 2020). Due to the variation in study methods, all meta-analyses were conducted using an inverse-variance random-effects model. Statistical heterogeneity between studies was examined using *I*^2^ and Chi^2^ statistics. The *I*^2^ statistic describes the variability in effect estimates that may be apportioned to study heterogeneity and is displayed as a percentage value where 0% to < 30% might not be important; 30% to < 60% may represent moderate heterogeneity; 60 to 90% may represent substantial heterogeneity, and > 90% may represent considerable heterogeneity [17]. Chi^2^ statistics were deemed statistically significant for heterogeneity when *p* < 0.1, although it is recommended to base analysis models on a thorough examination of heterogeneity rather than solely on one statistic [17].

Continuous outcome variable data were analysed by inputting each individual study’s mean outcome values, standard deviation (SD) and sample size for the PHP and control groups. The mean difference between groups and 95% confidence interval (CI) were calculated and a weighted pooled estimate for the individual studies was obtained. Dichotomous outcome variable data were analysed by inputting each individual study’s number of events and sample size for the PHP and control groups. The odds ratio (OR) and 95% CI were calculated, and the inverse variance method applied in order to determine the weighted pooled estimate. Where a study reported no events in both groups, a continuity correction was used based on a function of the reciprocal of the opposite group [18].

For meta-analyses that included both studies that used blinded assessors and studies that used unblinded assessors (i.e. assessors were aware whether or not participants had PHP), sensitivity analyses were performed to assess for potential assessor bias where appropriate. Where studies reported unilateral foot data and where the symptomatic foot of the PHP participants could be compared to the same side in the control group (i.e. left vs left or right vs right), then the most conservative data were used for the purpose of meta-analysis.

## Results

### Study characteristics

**Fig. 1:**
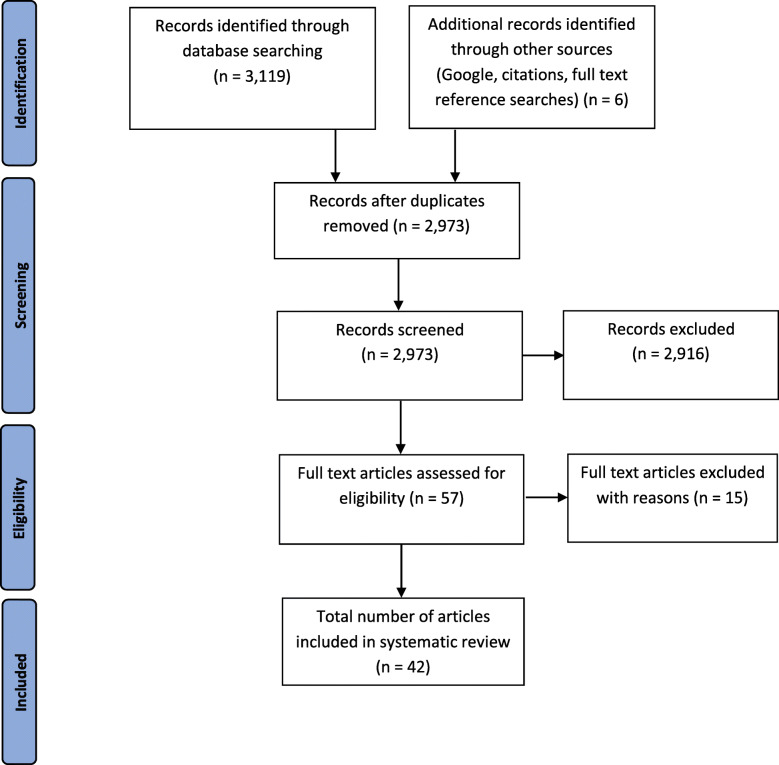
Study flow diagram

The database search identified a total of 2973 unique citations of which 42 studies met the eligibility criteria for inclusion in the review [19–60] – Fig. 1. The excluded studies and the reasons for exclusion following full text article assessment are presented in Additional File 2. There was a total sample size of 2928 participants; 1367 PHP participants (62% female, mean age 46 years) and 1561 control participants (56% female, mean age 42 years).

### Quality appraisal and risk of bias

Overall, 16 of the 42 (38%) studies reported if assessors were blinded to whether participants had or did not have PHP, BMI was not recorded in 21 (50%) studies, bilateral heel data (where participants’ had PHP on both feet and/or where both asymptomatic feet of the controls were included) was included in 25 (60%) studies, and the population from which the sample was recruited was not recorded in 23 (56%) studies – Table 1.

**Table 1:** Study and participant characteristics

Study ID	Imaging modality	Sample size		Blinding	Uni or bilateral	Sample type	Female %		Mean age (years)		Mean BMI (kg/m^**2**^)	
		PHP	Control				PHP	Control	PHP	Control	PHP	Control
Aggarwal 2020	Ultrasound	44	50	NR	Bilateral	NR	95.5	50.0	36.0	38.2	28.8	25.7
Akfirat 2003	Ultrasound/Radiograph	25	15	NR	Bilateral	NR	92.0	73.3	47.5	46.5	27.2, 28.4 ^1^	28.0
Berkowitz 1991	MRI	8	10	NR	Bilateral	NR	87.5	50.0	43.0	41.0	NR	NR
Bygrave 1998	Ultrasound	14	11	NR	Bilateral	NR	50.0	63.6	NR	NR	28.9	24.6
Cardinal 1996	Ultrasound	15	15	Unblinded	Bilateral	NR	60.0	NR	43.0	NR	NR	NR
Cetin 2001	Scintigraphy/Radiograph	22	17	NR	Unilateral	NR	77.3	52.9	47.4	53.3	29.2	28.7
Chen 2013	Ultrasound	38	21	NR	Unilateral	Community	63.2	42.9	45.2	45.1	25.4	23.3
Cheng 2012	Ultrasound	11	26	Blinded	Bilateral	NR	45.5	53.8	NR	NR	NR	NR
Cheung 2016	MRI	10	10	NR	Unilateral	Athletic	50.0	50.0	32.6	34.5	NR	NR
Fabrikant 2011	Ultrasound	30	33 ^2^	NR	Bilateral	Community	53.3	54.5	57.1	58.6	32.1	28.3
Fernandez-Lao 2016	Ultrasound	22	22	NR	Unilateral	NR	50.0	50.0	47.9	47.2	NR	NR
Finkenstaedt 2018	MRI	22	15	Blinded	Unilateral	NR	68.2	80.0	54.0	47.0	28.8	23.7
Gatz 2020	Ultrasound/Sonoelastography	31	10	Blinded	Bilateral	Community	74.2	50.0	48.9	30.4	26.7	22.8
Genc 2005	Ultrasound	30	30 ^2^	Unblinded	Bilateral	NR	90.0	90.0	43.1	42.9	28.1	28.3
Gibbon 1999	Ultrasound	190	48	Unblinded	Bilateral	Community	43.2	58.3	53.0	48.0	NR	NR
Granado 2018	Ultrasound	20	20 ^3^	Unblinded	Unilateral	NR	65.0	13.0	47.0	43.0	28.3	25.3
Hogan 2020	Ultrasound	16	16	Unblinded	Unilateral	Community	81.3	81.3	26.1	25.0	NR	NR
Kamel 2000	Ultrasound	20	20	NR	Bilateral	NR	55.0	55.0	NR	NR	NR	NR
Karabay 2007	Ultrasound	23	23	NR	Bilateral	NR	65.2	47.8	NR	NR	NR	NR
Lee 2014	Sonoelastography	13	15	Unblinded	Bilateral	NR	NR	NR	45	46.0	NR	NR
Lin 2015	Sonoelastography	16	20	NR	Unilateral	Community	56.3	50.0	51.8	25.5	24.6	23.6
McMillan 2013	Ultrasound	30	30	Unblinded	Unilateral	Community	50.0	50.0	57.0	57.0	31.0	29.0
Osborne 2006	Radiograph	21	78	Blinded	Bilateral	NR	NR	NR	51.8	43.4	NR	NR
Ozdemir 2005	Ultrasound	39	22	Blinded	Bilateral	Community	74.4	63.6	45.0	36.0	28.0	25.0
Prichasuk 1994	Radiograph	82	400	Unblinded	Bilateral	Community	90.2	50.0	46.1	NR	NR	NR
Rios-Diaz 2015	Sonoelastography	21	23	Blinded	Unilateral	NR	14.3	47.8	38.0	23.7	26.5	23.3
Rome 2002	Ultrasound	33	64 ^4^	Blinded	Unilateral	Mixed	NR	NR	24.6	23.9	23.1	22.3
Sabir 2005	Ultrasound/MRI	77	77	Blinded	Bilateral	NR	85.7	81.8	45.9	42.0	34.2	25.2
Sahin 2010	Radiograph	42	40	Unblinded	Bilateral	Community	76.2	75.0	48.0	47.2	NR	NR
Schillizzi 2020	Ultrasound/Sonoelastography	17	20	Unblinded	Bilateral	NR	NR	NR	50.5	47.5	25.0	24.0
Sconfienza 2013	Ultrasound/Sonoelastography	80	50	Blinded	Unilateral	Community	46.3	46.0	46.3	44.3	NR	NR
Song 2019	MRI	18	19	NR	Bilateral	NR	61.1	47.3	45.6	40.8	NR	NR
Sutera 2010	MRI	20	20	Blinded	Unilateral	NR	20.0	30.0	36.0	33.0	NR	NR
Tsai 2000	Ultrasound	102	33	Blinded	Bilateral	NR	69.6	51.5	45.0	41.1	24.5, 25.3 ^1^	23.3
Turgut 1999	Radiograph	73	120	Blinded	Bilateral	Community	69.9	NR	47.0	NR	NR	NR
Wall 1993	Ultrasound	19	20	Blinded	Unilateral	NR	47.4	50.0	49.2	45.5	NR	NR
Walther 2004	Ultrasound	20	20	NR	Unilateral	NR	80.0	60.0	45.0	42.0	NR	NR
Wearing 2007	Ultrasound	10	10	Unblinded	Unilateral	NR	70.0	70.0	48.0	47.0	NR	NR
Wearing 2010	Ultrasound	9	9	Blinded	Unilateral	Community	66.7	66.7	48.0	46.0	29.0	28.9
Williams 1987	Scintigraphy ^5^	45	NR	Blinded	Bilateral	Community	44.4	NR	57.5	NR	NR	NR
Wu 2011	Ultrasound/Sonoelastography	13	20 ^6^	Unblinded	Bilateral	Community	53.8	50.0	49.5	55.4	23.5	23.1
Wu 2015	Sonoelastography	20	30	Blinded	Bilateral	Community	60.0	63.3	45.1	41.6	22.5, 21.5 ^1^	22.2

Notes: ^1^ Study reported unilateral and bilateral data, respectively; ^2^ Left-sided PHP and control group data extracted for meta-analysis; ^3^ Right-sided PHP and control group data extracted for meta-analysis; ^4^ Matched control group data; ^5^ Radiograph data from this study were excluded (see Additional file 2); ^6^ Data reported for older age group of two control groups; NR = Not reported

All 42 studies were appraised using the NIH quality assessment tool, with 16 (38%) rated poor, 17 (41%) rated fair, and 9 (21%) rated good (Table 2). Details of the quality appraisal for each study are included in Additional file 3.

**Table 2:**
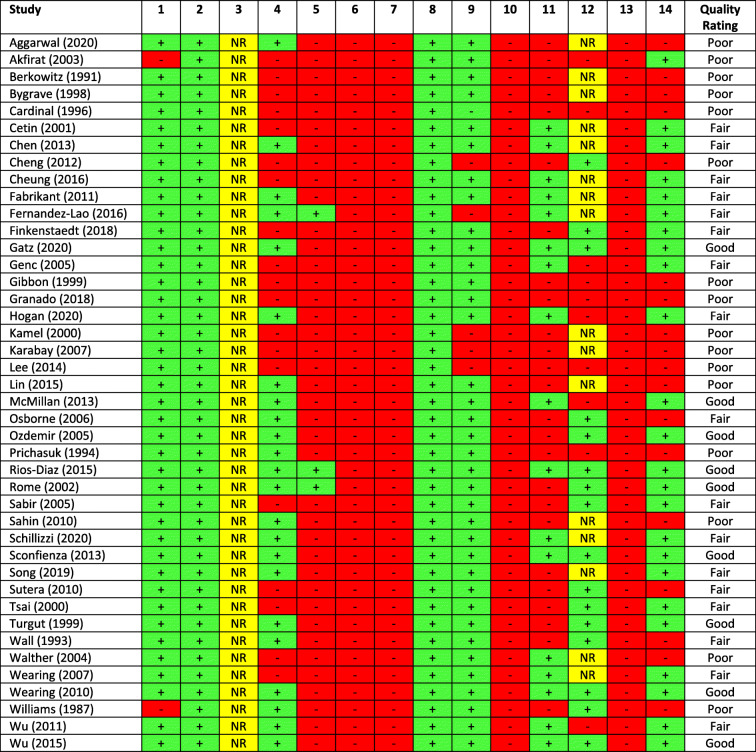
Quality appraisals (heading numbers represent question numbers in the NIH appraisal tool [16]

### Plantar fascia thickness

Measurements of plantar fascia thickness were reported in 31 studies, and of these, 26 used ultrasound alone [19–44], 4 used MRI alone [46–49], and 1 used ultrasound and MRI [45].

#### Ultrasound

Twenty-seven studies measured plantar fascia thickness using ultrasound, 21 of which were considered appropriate for meta-analysis. Of the six studies that were excluded, a single study measured maximal thickness rather than proximal thickness and therefore could not be combined for meta-analysis [28], one study did not report the SD of the mean thickness [30], one study reported the number of plantar fascia thicker than 4 mm rather than mean plantar fascia thickness [38], one study reported the median thickness [24], one study purposively sampled for plantar fascia thickness > 5 mm (i.e. participants were only eligible if their fascia was > 5 mm thick) [41], and it was unclear in one study what the group sizes were for either the left or right foot data in the PHP group [19]. Of the 21 studies included in the meta-analysis, only 7 reported that the assessors were blinded to whether participants had or did not have PHP [25, 36, 37, 39, 40, 43, 45].

**Fig. 2:**
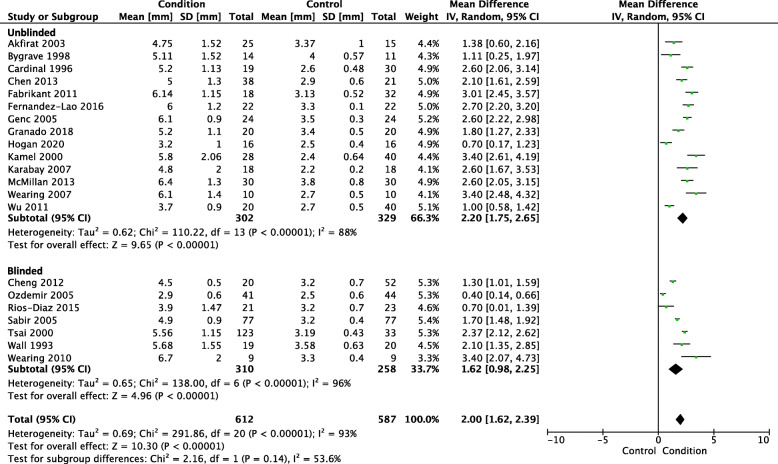
Ultrasound plantar fascia thickness

Meta-analysis of the 21 studies that reported ultrasound measurements of plantar fascia thickness included a total of 612 PHP participants and 587 control participants. PHP participants had a mean plantar fascia thickness that was 2.00 mm (95% CI 1.62 to 2.39) thicker than control participants (*p* < 0.001) – see Fig. 2. Heterogeneity was found to be considerable (Tau^2^ = 0.69; Chi^2^ = 291.86, *I*^2^ = 93%) for this meta-analysis.

A sensitivity analysis of the 7 studies that used blinded assessors was conducted (PHP *n* = 310, control *n* = 258). Compared with the overall analysis (presented above), the sensitivity analysis of studies that used blinded assessors found PHP participants to have a lower mean plantar fascia thickness of 1.62 mm (95% CI 0.98 to 2.25) thicker than control participants (*p* < 0.001) – see Fig. 2. This finding was also lower than the analysis of studies that used unblinded assessors (PHP *n* = 302, control *n* = 329), which found PHP participants to have a mean plantar fascia thickness of 2.20 mm (95% CI 1.75 to 2.65) thicker than control participants (*p* < 0.001). Heterogeneity was found to be considerable for the blinded analysis (Tau^2^ = 0.65; Chi^2^ = 138.00, *I*^2^ = 96%) and substantial for the unblinded analysis (Tau^2^ = 0.62; Chi^2^ = 110.22, *I*^2^ = 88%).

#### MRI

Five studies measured plantar fascia thickness using MRI [45–49], two of which used assessors that were blinded [45, 49]. Two of the studies measured proximal plantar fascia thickness [45, 49] and were included in a meta-analysis, whilst three of the studies measured at the point of maximal plantar fascia thickness and were included in a separate meta-analysis [46–48].

**Fig. 3:**
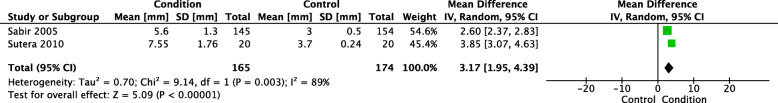
MRI proximal plantar fascia thickness

Meta-analysis of the two studies that measured proximal plantar fascia thickness included a total of 165 PHP participants and 174 control participants [45, 49]. PHP participants had a mean plantar fascia thickness that was 3.17 mm (95% CI 1.95 to 4.39) thicker than control participants (*p* < 0.001) – see Fig. 3. Heterogeneity was found to be substantial for this analysis (Tau^2^ = 0.70; Chi^2^ = 9.14, *I*^2^ = 89%).

**Fig. 4:**
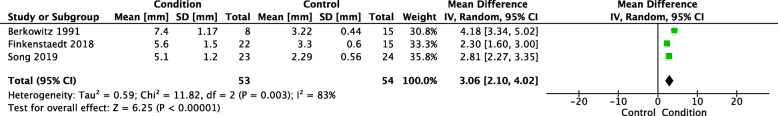
MRI maximal plantar fascia thickness

Meta-analysis of the three studies that measured maximal plantar fascia thickness included a total 53 PHP participants and 54 control participants [46–48]. PHP participants had a mean plantar fascia thickness that was 3.06 mm (95% CI 2.10 to 4.02) thicker than control participants (*p* < 0.001) – Fig. 4. Heterogeneity was found to be substantial for this analysis (Tau^2^ = 0.59; Chi^2^ = 11.82, *I*^2^ = 83%).

### Plantar fascia thickness > 4 mm

**Fig. 5:**
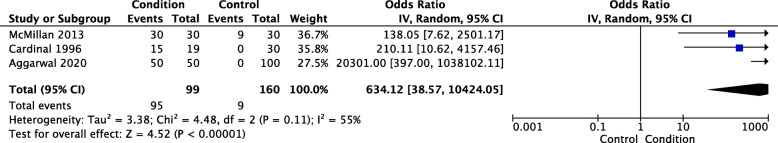
Plantar fascia thickness > 4 mm

Three unblinded ultrasound studies reported the number of participants with plantar fascia thickness > 4 mm [19, 22, 35]. Meta-analysis was conducted and included a total of 99 PHP participants and 160 control participants. PHP participants were greater than 600 times more likely to have a plantar fascia thickness > 4 mm compared with control participants (OR 634.12, 95% CI 38.57 to 10,424.05, *p* < 0.001) – see Fig. 5. Heterogeneity was found to be moderate for this analysis (Tau^2^ = 3.38; Chi^2^ = 4.48, *I*^2^ = 55%). One other ultrasound study reported a different cut-off for plantar fascia thickness of > 4.5 mm [38], and therefore was not included in the meta-analysis. It found 73 (91%) of the PHP participants had plantar fascia thickness > 4.5 mm compared to 4 (2%) of the control participants.

One MRI study reported the number of people with plantar fascia thickness > 4 mm [49]; this study found 15 (75%) of the PHP participants and none (0%) of the control participants presented with this finding.

### Plantar fascia tissue changes

#### Ultrasound hypoechogenicity

**Fig. 6:**
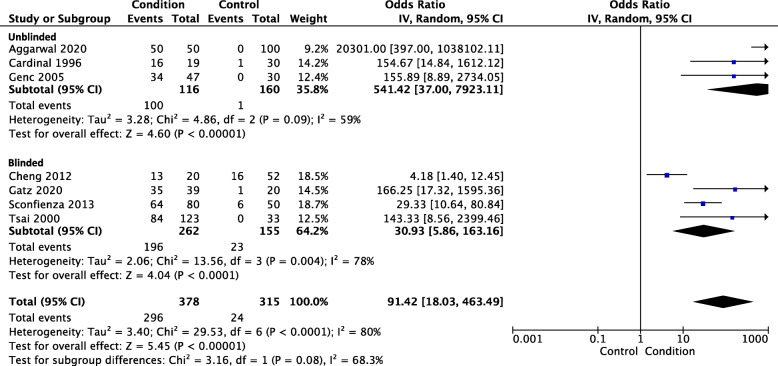
Ultrasound hypoechogenicity

Ten studies measured plantar fascia hypoechogenicity using ultrasound, and seven of these reported the presence or absence of hypoechogenicity (i.e. ‘yes’ or ‘no) and were appropriate for meta-analysis [19, 22, 25, 28, 29, 38, 39]. Two studies were excluded as they did not report the presence of a hypoechogenic signal in the control group [30, 36] and one study reported grade (1–4) of hypoechogenicity [37]. Meta-analysis of the seven studies included 378 PHP participants and 315 control participants. PHP participants were greater than 90 times more likely to present with hypoechogenic signal in the plantar fascia than control participants (OR 91.42, 95% CI 18.03 to 463.49, *p* < 0.001) – see Fig. 6. Heterogeneity was found to be substantial for this analysis (Tau^2^ = 3.40; Chi^2^ = 29.53, *I*^2^ = 80%).

A sensitivity analysis was conducted with four studies that used blinded assessors (PHP *n* = 262, control *n* = 155). Compared with the overall analysis (presented above), the sensitivity analysis of studies that used blinded assessors found lower odds of having hypoechogenicity in the PHP participants (OR 30.93, 95% CI 5.86 to 163.16, *p* < 0.001) – see Fig. 6. This finding was also lower than the analysis of studies that used unblinded assessors (OR 541.42, 95% CI 37.00 to 7923.11, *p* < 0.001). Heterogeneity was found to be substantial for the blinded analysis (Tau^2^ = 2.06; Chi^2^ = 13.56, *I*^2^ = 78%) and moderate for the unblinded analysis (Tau^2^ = 3.28; Chi^2^ = 4.86, *I*^2^ = 59%).

#### MRI signal hyperintensity

**Fig. 7:**
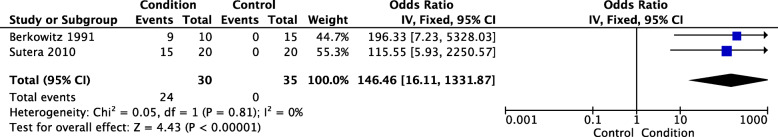
MRI hyperintensity

Two studies measured hyperintensity of the signal related to the plantar fascia using MRI [46, 49] and were appropriate for meta-analysis. A total of 30 PHP participants and 35 control participants were included in the analysis. PHP participants were greater than 140 times more likely to present with hyperintensity of the signal than control participants (OR 146.46, 95% CI 16.11 to 1331.87, *p* < 0.001) – see Fig. 7. Heterogeneity was found to be not important for this analysis (Tau^2^ = 0.00; Chi^2^ = 0.05, *I*^2^ = 0%).

### Plantar fascia hyperaemia

Three studies measured hyperaemia using power Doppler ultrasonography [23, 35, 41]. A meta-analysis could not be conducted due to heterogeneity between studies (e.g. variation in study design and outcome measures). One study purposively sampled a PHP group with fascia thickness > 5 mm [41]. Two studies graded hyperaemia using a 1–4 scale [35, 41], and a comparison of the number of people with hyperaemia between PHP and control participants in these two studies is presented in Table 3. The third study [23], measured vascular index using power Doppler ultrasonography with increased vascularity in the PHP group (mean 2.4, SD 1.4) compared to the control group (mean 1.6, SD 0.4).

**Table 3:** Comparison of plantar fascia hyperaemia classifications for PHP and control groups as measured by power Doppler ultrasound (hyperaemia graded from 1 to 4^†^)

Study	Group	Grade 1	Grade 2	Grade 3	Grade 4
		Count (%)	Count (%)	Count (%)	Count (%)
McMillan et al., 2013 [35]	PHP group (*n* = 30)	22 (73)	5 (17)	2 (7)	1 (3)
	Control group (n = 30)	28 (93)	2 (7)	0 (0)	0 (0)
Walther et al., 2004 [41]	PHP group (*n* = 20)	10 (50)	2 (10)	5 (25)	3 (15)
	Control group (n = 20)	19 (95)	1 (5)	0 (0)	0 (0)

^†^ Grading scale: 1 represented normal tissue perfusion, 2 mild hyperaemia, 3 moderate hyperaemia, and 4 marked hyperaemia with a confluent surrounding vascular blush

### Plantar fascia elasticity

Seven studies measured elasticity of the plantar fascia using sonoelastography [24, 28, 37, 38, 44, 50, 51]. Two studies excluded symptomatic participants with abnormal features on standard ultrasound [50, 51]. A meta-analysis could not be conducted due to heterogeneity between the studies (e.g. study design and sonoelastographic variables measured). These studies generally found that the plantar fascia was softer or less stiff. A summary of individual study results is presented in Table 4.

**Table 4:** Summary of findings for individual sonoelastography studies

Study	Sample size	Findings
Gatz et al., 2020 [28]	PHP = 39, Control = 20	PHP participants had significantly lower Young’s modulus values at the fascia insertion (mean 46.3 kPa, SD 5.5) compared to control participants (mean 87.6 kPa, SD 22.6).
Lee et al., 2014 [50]	PHP = 18, Control = 18	16 (89%) PHP participants had the presence of plantar fascia softening compared to only 9 (59%) of the control participants.
Rios-Diaz et al., 2015 [37]	PHP = 21, Control = 23	72.6% of fascias were of intermediate stiffness with no association with PHP.
Schillizzi et al., 2020 [24]	PHP = 19, Control = 20	PHP participants had significantly lower shear wave velocity expressed in meters/second (SWV m/s) (median 3.8 m/s, IQR 1.5 to 5.1) compared to control participants (median 5.1 m/s, IQR 3.0 to 6.9).
Sconfienza et al., 2013 [38]	PHP = 80, Control = 50	PHP participants’ fascia were less elastic than control participants’ fascia (median elasticity values 11 and 7, respectively, where a higher score indicates less elasticity).
Wu et al., 2011 [44]	PHP = 13, Control = 40	PHP participants had significantly less red (hard) pixel intensity (measured on a scale from 0 to 255) in the fascia compared to older control participants (mean 133.7, SD 13.4 compared with mean 147.8, SD 10.3, respectively).
Wu et al., 2015 [51]	PHP = 30, Control = 30	Participants with unilateral PHP had significantly less red (more elastic) pixel intensity (range 0–255) compared to control participants (mean 127.1, SD 7.4 to mean 146.9, SD 9.1, respectively).

Notes: kPa: Kilopascal, SWV m/s: Shear wave velocity expressed in meters/second, IQR: Interquartile range, SD: Standard deviation

### Plantar fascia tear

Six studies recorded the presence of plantar fascia tears [19, 20, 22, 34, 45, 49]. Four studies used ultrasound alone [19, 20, 22, 34], one study used MRI alone [49], and one used both ultrasound and MRI [45].

#### Ultrasound

**Fig. 8:**
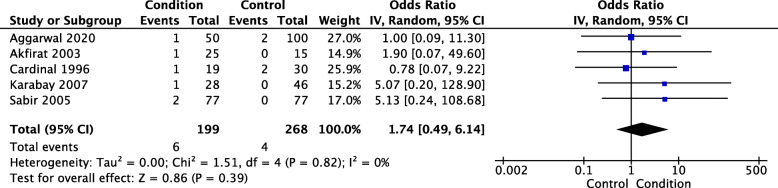
Ultrasound plantar fascia tear

Five studies recorded the presence of plantar fascia tears using ultrasound [19, 20, 22, 34, 45]. Only one of the studies included assessors that were blinded [45]. Meta-analysis of all five studies was conducted with a total of 199 PHP participants and 268 control participants. PHP participants were almost two times more likely to have a plantar fascia tear than control participants, but this was not statistically significant (OR 1.74, CI 0.49 to 6.14, *p* = 0.390) – see Fig. 8. Heterogeneity was found to be not important for this analysis (Tau^2^ = 0.00; Chi^2^ = 1.51, *I*^2^ = 0%).

#### MRI

**Fig. 9:**
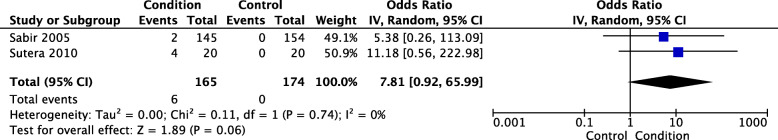
MRI plantar fascia tear

Two studies reported the presence of plantar fascia tears using MRI [45, 49]. Only one of the studies included assessors that were blinded [45]. Meta-analysis of the two studies was conducted with a total of 165 PHP participants and 174 control participants. PHP participants were almost 8 times more likely to present with a plantar fascia tear than control participants, but this was not statistically significant (OR 7.81, 95% CI 0.92 to 65.99, *p* = 0.060) – see Fig. 9. Heterogeneity was found to be not important for this analysis (Tau^2^ = 0.00; Chi^2^ = 0.11, *I*^2^ = 0%).

### Heel fat pad thickness

Five studies measured heel fat pad thickness [21, 34, 39, 45, 53], one of which reported measurements using both ultrasound and MRI [45].

#### Ultrasound

Heel fat pad thickness measurements were reported in five studies that used ultrasound [21, 34, 39, 45, 53], three of which included assessors that were blinded [39, 45, 53]. Three studies reported unloaded heel fat pad measurements [34, 39, 45] and were appropriate for meta-analysis. The remaining two studies reported loaded heel fat pad measurements [21, 53] and were appropriate for a separate meta-analysis.

**Fig. 10:**
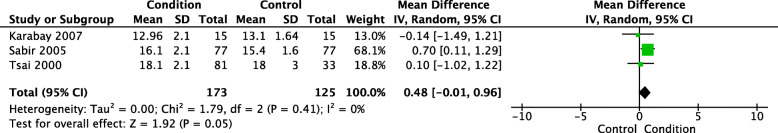
Unloaded heel pain pad thickness

Meta-analysis of the three studies that measured unloaded heel fat pad thickness included 173 PHP participants and 125 control participants. PHP participants had a mean unloaded fat pad thickness that was 0.48 mm thicker (95% CI − 0.01 to 0.96) than control participants, but this was not statistically significant (it approached significance *p* = 0.050) – see Fig. 10. Heterogeneity was found to be not important for this analysis (Tau^2^ = 0.00; Chi^2^ = 1.79, *I*^2^ = 0%).

**Fig. 11:**
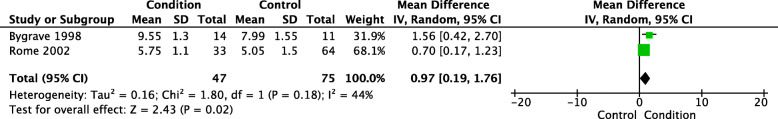
Loaded heel fat pad thickness

Meta-analysis of the two studies that measured loaded heel fat pad thickness included 47 PHP participants and 75 control participants. PHP participants had a mean loaded fat pad thickness that was 0.97 mm thicker (95% CI 0.19 to 1.76) than control participants, which was statistically significant (*p* = 0.020) – see Fig. 11. Heterogeneity was found to be moderate for this analysis (Tau^2^ = 0.16; Chi^2^ = 1.80, *I*^2^ = 44%).

#### MRI

One study reported unloaded heel fat pad thickness on MRI [45]. PHP participants had a mean unloaded fat pad thickness that was 0.5 mm thicker than control participants (*p* < 0.001). The PHP participants had a fat pad that was 17.6 mm (SD 2.6 mm) thick and the control participants had a fat pad that was 17.1 mm (SD 1.6 mm) thick.

### Plantar intrinsic muscle size

Two studies measured muscle size; one study measured cross-sectional area and muscle thickness of the abductor hallucis muscle using ultrasound [31], and one study measured intrinsic foot muscle volume, which was normalised to body mass using MRI [54].

The ultrasound study found no significant difference (*p* = 0.45 – the authors presented *p*-values to 2 decimal places only) in abductor hallucis muscle cross-sectional area between the PHP participants (mean 2.00 cm^2^, SD 0.52) and the control participants (mean 1.87 cm^2^, SD 0.47). There was also no significant difference (*p* = 0.46) in abductor hallucis muscle thickness between the PHP participants (mean 1.16 cm, SD 0.23) and the control participants (mean 1.10 cm, SD 0.24) [31].

The MRI study measured muscle volume in three areas; total intrinsic foot muscle volume, rearfoot muscle volume, and forefoot muscle volume (all of which were normalised to body mass) [54]. Firstly, PHP participants had 245.3 mm^3^/kg less total intrinsic foot muscle volume compared to control participants. PHP participants had a mean of 1838.0 mm^3^/kg (SD 277.1) and control participants had a mean of 2083.3 mm^3^/kg (SD 258.7). This difference was not statistically significant (it approached significance *p* = 0.056), but the Cohen’s *d* effect size was large at 0.92. Secondly, PHP participants had 195.5 mm^3^/kg less rearfoot volume compared to control participants. PHP participants had a mean of 746.8 mm^3^/kg (SD 129.18) and control participants had a mean volume of 942.5 mm^3^/kg (SD 208.02). This difference was statistically significant (*p* = 0.023) and the Cohen’s *d* effect size was large at 1.13. Thirdly, PHP participants had 49.6 mm^3^/kg less forefoot volume than control participants. PHP participants had a mean volume of 1091.2 mm^3^/kg (SD 169.51) and the control participants had a of 1140.8 mm^3^/kg (SD 149.48). This difference was not statistically significant (*p* = 0.496) and the Cohen’s *d* effect size was moderate at 0.31.

### Calcaneal spur

**Fig. 12:**
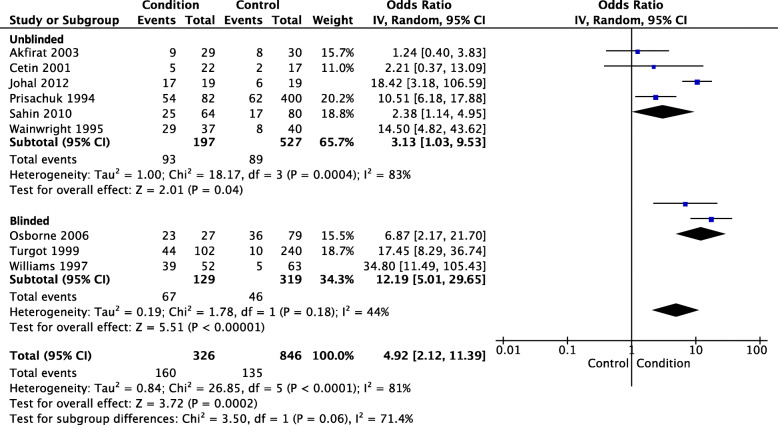
Calcaneal spur

Six studies measured the presence of calcaneal spur using x-ray [20, 55–59]. Only two of the studies used assessors that were blinded [56, 59]. Meta-analysis of the six studies was conducted with a total of 326 PHP participants and 846 control participants. PHP participants were almost 5 times more likely to present with a calcaneal spur compared to control participants (OR 4.92, 95% CI 2.12 to 11.39, *p* < 0.001) – see Fig. 12. Heterogeneity was found to be substantial for this analysis (Tau^2^ = 0.84; Chi^2^ = 26.85, *I*^2^ = 81%).

A sensitivity analysis of the two studies that used blinded assessors was conducted (PHP *n* = 129, control *n* = 319). Compared with the overall analysis (presented above), the sensitivity analysis of studies that used blinded assessors found greater odds of having a calcaneal spur in the PHP participants (OR 12.19, 95% CI 5.01 to 29.65, *p* < 0.001) – see Fig. 12. This finding was also higher than the analysis of studies that used unblinded assessors (OR 3.13, 95% CI 1.03 to 9.53, *p* = 0.040). Heterogeneity was found to be moderate for the blinded analysis (Tau^2^ = 0.19; Chi^2^ = 1.78, *I*^2^ = 44%) and substantial for the unblinded analysis (Tau^2^ = 1.00; Chi^2^ = 18.17, *I*^2^ = 83%).

### Bone marrow oedema

Two studies measured the presence of bone marrow oedema within the calcaneus using MRI [46, 49], with one study using assessors that were blinded [49]. A meta-analysis was not conducted as there were no events in either the PHP or control group in one study [46]. The other study found that 7/20 PHP participants (35%) presented with bone marrow oedema in the calcaneus compared to 0/20 (0%) in control participants [49].

### Calcaneal crescent sign

One study measured the cross-sectional area and volume of the calcaneal tuberosity trabeculae (calcaneal crescent) using MRI [47]. This study found that PHP participants had greater cross-sectional area of the calcaneal crescent when compared with control participants (mean 100.2, SD 29.2 versus mean 73.7, SD 18.9 mm^2^, *p* = 0.019), greater volume, (mean 3.06, SD 1.10 versus mean 1.99, SD 0.68 cm^3^, *p* = 0.006), and lower contrast-to-noise ratio (mean − 38.1, SD 11.0 versus mean − 28.4, SD 13.0, *p* = 0.009).

### Calcaneal radioisotope uptake

Two studies measured radioisotope uptake in the calcaneus using technetium-99 m bone scans [55, 60]. A meta-analysis was not conducted as one study did not report the phase of the bone scan that observations were recorded [55], and the other study did not report the number of control participants [60]. One study reported increased uptake in the calcaneus in 16/22 (73%) in PHP participants and 0/17 (0%) in control participants [55]. The other study reported increased uptake in 31/52 (60%) of PHP participants and 0% (number not reported) in control participants [60]. In both studies, no statistical comparisons of the radioisotope uptake differences between the groups were made.

## Discussion

The aim of this systematic review was to synthesise medical imaging features associated with PHP. The review included 42 studies, which is an increase of 19 additional studies compared with our previous review more than a decade ago [14]. Meta-analyses of multiple studies found several imaging features associated with PHP including a thickened plantar fascia, abnormalities of the plantar fascia including the presence of fascia tears, abnormalities of adjacent tissue such as the heel fat pad, and calcaneal spurs. These imaging features depict a painful condition affecting the plantar fascia, surrounding soft tissue structures, and bone. Meta-analyses could not be conducted for several imaging features due to variation in methods, but individual studies found hyperaemia of the plantar fascia, reduced fascia elasticity, intrinsic foot muscle atrophy, increased calcaneal radioisotope uptake, and calcaneal bone marrow oedema were also associated with PHP, however these all require further investigation.

### Interpretation of findings

The imaging features outlined above are consistent with degenerative soft tissue changes characterised by fascia thickening, fascia tissue changes, presence of fascia tears, and loss of fascia elasticity. A thickened plantar fascia with degenerative changes is consistent with our previous systematic review [14].

Regarding plantar fascia thickness, meta-analysis of ultrasound studies found that participants with PHP had a mean proximal plantar fascia thickness that was 2.00 mm thicker than control participants. However, a sensitivity analysis found a lesser but still thicker difference of 1.62 mm for blinded studies compared to 2.20 mm for the unblinded studies. This suggests that unblinded studies, with a higher risk of assessor bias, may have over-estimated plantar fascia thickness in participants with PHP. Therefore, we have elected to focus on the more conservative interpretation that people with PHP have a plantar fascia that is 1.62 mm thicker on average than people without PHP (we have done this for all other findings in the discussion below). Meta-analysis of MRI studies found that participants with PHP had an even thicker plantar fascia (3.17 mm thicker) than control participants; although, there were only 2 studies in the MRI analysis compared with 21 studies in the ultrasound analysis. MRI thickness measurements can over-estimate tissue thickness measurements as it is dependent on the orientation of the slice from which the measurement is taken, and if that slice is oblique to the plane of maximum thickness, it can measure the tissue to be thicker than it actually is. This has been found in other populations and elsewhere in the body [61]. Accordingly, ultrasound measurements of tissues like the plantar fascia are generally more accurate.

Plantar fascia thickness changes can also be categorised by dichotomising participants into those with a plantar fascia that is thicker than 4 mm and those with a plantar fascia that is 4 mm or less [10]. Meta-analysis revealed that participants with PHP were 634 times more likely to have a plantar fascia thickness greater than 4 mm when compared with healthy controls. However, this finding should be interpreted with the knowledge that all studies in the analysis used assessors that were not blinded, and that two [19, 22] of the three studies used paired heel data from the same participants if they had bilateral PHP. Paired data can be used to increase sample size, however it can lead to reduced variability in the sample and result in statistically significant findings that may be spurious [62]. Nevertheless, it can be concluded that the plantar fascia is thicker in people with PHP on both ultrasound and MRI, and the odds of the fascia being thicker than 4 mm is greatly increased on ultrasound.

Not only does the fascia thickness increase in people with PHP, but tissue changes within the fascia can also be detected with medical imaging. The presence of plantar fascia hypoechogenicity on ultrasound and hyperintensity of the signal on MRI were found to be significantly associated with PHP. Participants with PHP were nearly 31 times more likely to have hypoechogenicity on ultrasound and 146 times more likely to have signal hyperintensity on MRI of the plantar fascia. Accordingly, people with PHP are substantially more likely to show signs that are consistent with degeneration of the plantar fascia on ultrasound and MRI as detected by hypoechogenicity and hyperintensity, respectively.

In addition to the plantar fascia tissue changes outlined above, we were interested in whether plantar fascia tears were more likely in people with PHP, which was not analysed in our previous review [14]. Meta-analysis found no significant differences between people with and without PHP for the presence of a plantar fascia tear on both ultrasound and MRI. However, both meta-analyses had relatively low sample sizes, and as a consequence, the OR estimates had wide confidence intervals, so more studies are needed for this analysis to improve the precision of the estimates, which is needed to know definitively if plantar fascia tears are truly associated with PHP. In addition, studies included in this analysis provided unclear definitions of a tear on imaging, and some may have assessed for a full tear only, as opposed to both partial and full tears. A tear within the fascia, whether partial or full, is of clinical interest, as it may correspond to an acute episode where the patient remembers an incident that triggered the pain and is worth considering during treatment as greater weightbearing relief may be necessary for healing to occur. We believe this imaging feature needs further investigation with a strict definition of what constitutes a tear.

While there is clear evidence for changes in the plantar fascia tissue in people with PHP on ultrasound or MRI, such as thickness or structural changes, findings from some other modalities are less convincing at this stage. Sonoelastography studies included in this review suggest a loss of elasticity in the fascia in those with PHP. Two of the studies reported this feature occurring in isolation without other plantar fascia changes [50, 51], which suggests there might be the potential for early diagnostic ability with sonoelastography, however it is currently unknown whether such a finding is clinically worthwhile from a management perspective. A meta-analysis could not be conducted due to differences between studies in the sonoelastographic variables measured, therefore findings from sonoelastography studies could not be synthesised or summarised. Despite sonoelastography being of interest in PHP research, future studies of PHP using sonoelastography need improvement; that is, methods and measurements need to be standardised.

There may also be differences in plantar intrinsic muscle size between PHP and healthy controls, but again, the lack of studies prohibited a meta-analysis of this. Indeed, in two studies, intrinsic foot muscle size (cross-sectional area and volume) was found to be decreased in participants with PHP. Our findings are essentially the same as those of Osborne and colleagues [63] who conducted a systematic review that was specific to muscle strength and size in people with and without PHP; that is, they did not investigate wider medical imaging findings. One issue when considering muscle size from cross-sectional studies is that causality cannot be inferred, so even if people with PHP have smaller intrinsic muscles, for example, it cannot be determined if the decrease in size is the cause of PHP or a result of PHP [63]. It is plausible, though, that the pain associated with PHP limits function, and as a consequence, muscle size decreases due to atrophy, so this is likely a secondary finding of PHP. However, such a finding helps inform whether muscle atrophy is indeed present with PHP, which may lead to further studies to more rigorously investigate its clinical relevance.

Hyperaemia is the active engorgement of vascular structures and is one of the primary responses to inflammatory stimuli. A meta-analysis of studies that measured hyperaemia in this review was not appropriate due to methodological heterogeneity between studies, however there was evidence from two studies of hyperaemia being more frequent in participants with PHP. Further, the presence of severe hyperaemia was only found in participants with PHP and not in healthy control participants. However, the degree of hyperaemia detected was substantially less in one study [35] than the other study [41], so additional studies are needed to determine with certainty if hyperaemia is associated with PHP, and consequently, whether it is worthwhile evaluating the effectiveness of treatments aimed at optimising the healing process of injured connective tissue structures, such as prolotherapy [64].

Change in the thickness of the plantar heel fat pad has also been studied. People with PHP were found on ultrasound to have a mean loaded fat pad thickness that was 0.97 mm thicker that people without PHP. This is somewhat supported by the mean unloaded fat pad thickness that was 0.48 mm thicker, although this finding was not found to be statistically significant (it was almost statistically significant at *p* = 0.050). It is currently unknown if these values are clinically important, however a thicker fat pad may be an adaptive response to vertical load; such as prolonged standing, running or a high BMI, a mechanism that has similarly been proposed for calcaneal spur development [65, 66]. If so, using soft orthotic materials or shoe midsoles may dissipate increased force under the heel. Further, contoured orthoses will have a similar effect by decreasing force and plantar pressure under the heel [67, 68].

Several other imaging features relating to bone were also identified. People with PHP were more likely to have plantar calcaneal spurs, bone marrow oedema and increased radioisotope uptake in the calcaneus. In this review, meta-analysis revealed that PHP participants were greater than 5 times more likely to present with a plantar calcaneal spur than control participants, which is slightly lower than the finding in our previous review [14]. A sensitivity analysis of blinded studies found that PHP participants were 12 times more likely to have a plantar calcaneal spur when compared to control participants, which counterintuitively, was higher than the unblinded studies. However, the two blinded studies both used paired heel data, which as stated previously, may affect the independence of the sample and any subsequent statistical analysis. With this in mind, we have elected to focus on the findings from the overall analysis of all studies, which found that people with PHP were 5 times more likely to have a plantar calcaneal spur. Isolated plantar calcaneal spurs are known to frequently co-exist with plantar fascia changes [69], and as such, they are unlikely to represent a discrete clinical manifestation. They are also frequently found in people without PHP [70], are associated with increasing age and obesity, and may be a response to vertical load rather than longitudinal traction at the origin of the fascia at the plantar calcaneus [65], although this is still somewhat under debate [71]. Accordingly, the finding of increased odds of plantar calcaneal spurs in people with PHP is of interest, however it is an association only and unlikely to be the cause of pain. Further, the presence of a plantar calcaneal spur has limited relevance to treatment, unless the spur is fractured, in which case fracture management principles would be necessary [72]. The use of x-rays, therefore, has a limited place in PHP.

The presence of bone marrow oedema was not measured in our previous review [14]. Two studies included in this review measured the presence of bone marrow oedema, although they were not appropriate for meta-analysis. An MRI study that used blinded assessors found over one third of PHP participants had bone marrow oedema in the calcaneus [49]. Interestingly, there was a small sub-group of symptomatic participants with bone marrow oedema who had clinical symptoms of PHP but no abnormalities of the plantar fascia. A moderate association between bone marrow lesions, structural progression, and longitudinal change in pain has been reported in knee osteoarthritis [73]. The foot and ankle has had limited study compared to the knee [74], however bone marrow oedema may present with unique clinical symptoms in PHP such as night pain [75]. The aetiology of bone marrow oedema is still uncertain, but treatment usually involves analgesics and offloading the limb. Further, increased radioisotope uptake in the calcaneum of PHP participants in scintigraphy studies [55, 60] lends support to a subset of people with PHP who have increased metabolic bone turnover within the calcaneus. The exact physiological process for this condition is unclear, but it is likely to be load-related and represents a target for further evaluation to determine its clinical relevance. If such a condition is found to be definitively associated with PHP, then this may represent a fatigue or stress injury of the bone. Another study [47], found that PHP participants also had greater cross-sectional area of the calcaneal tuberosity trabeculae (calcaneal crescent sign), which supports a fatigue or stress injury hypothesis, or at least a response to bone stress. The lack of studies investigating this feature precludes a definitive statement on the relevance of the crescent sign at this stage, although if further studies support this finding, it would be in keeping with a bone stress phenomenon in PHP.

### Limitations

This systematic review was designed to be a comprehensive review of the literature, however its findings should be considered in relation to several limitations. Firstly, it is possible that some appropriate studies may not have been identified and included. As in our previous review [14], studies were only included if they reported medical imaging findings in adult participants with PHP and compared these findings with those from independent control participants who were asymptomatic of PHP. In doing so, 15 studies that did not meet these criteria were excluded and therefore, all imaging features associated with PHP may not have been included in this review. Secondly, there was substantial heterogeneity in most of the meta-analyses and only one-fifth of studies were rated ‘good’ on quality assessment. Over half the studies included bilateral heel data from the same participant, which could have affected the results of our meta-analyses, and a similar proportion did not report where the study sample was recruited from or participants’ BMI, which leads to generalisability concerns. The majority of the studies did not use assessors who were blinded to the status of the participants (i.e. whether or not they had PHP), which could have led to assessor bias. This may have over-estimated the associations or differences we found, however we tried to take a conservative approach to this issue and conducted sensitivity analyses where appropriate. While the extent of these associations changed depending on blinding, what did not change was whether an association existed. The majority of studies also did not report inter- and intra-assessor reliability for imaging observations, which may have affected the accuracy of the imaging observations made. Ideally, assessors should demonstrate both intra- and inter-assessor reliability, which is something future studies should determine prior to data collection. Lastly, some of the meta-analyses included only two studies, and relatively small sample sizes, so the precision of the estimates of the associations for these analyses may be less than ideal. Further studies investigating these associations should improve the precision of these estimates.

## Conclusions

This systematic review investigated medical imaging features associated with PHP. Meta-analyses found those with PHP were more likely to have a thickened plantar fascia on ultrasound and MRI (which is greater than 4 mm), abnormal plantar fascia tissue as detected by ultrasound hypoechogenicity or MRI hyperintensity, a thicker loaded plantar heel fat pad on ultrasound, and a plantar calcaneal spur on plain film x-ray. In addition, there is some evidence from more than one study for hyperaemia within the plantar fascia identified on power Doppler ultrasound and bony abnormalities within the calcaneus such as increased bone uptake on technetium scans and bone marrow oedema on MRI. Whilst these medical imaging features may aid with diagnosis, additional high-quality studies investigating medical imaging findings for some of these imaging features would be worthwhile to improve the precision of these findings and determine their clinical relevance.

## Supplementary Information

**Additional file 1.**

**Additional file 2.**

**Additional file 3.**

## Data Availability

The data analysed during this study are available from the corresponding author upon reasonable request.

## Abbreviations

BMI: Body mass index
CI: Confidence interval
MRI: Magnetic resonance imaging
NIH: National Institutes of Health
OR: Odds ratio
PHP: Plantar heel pain
SD: Standard deviation
