# Supplementary material for: Medical imaging for plantar heel pain: a systematic review and meta-analysis
Source: J Foot Ankle Res. 2022 Jan 22;15:4. doi: 10.1186/s13047-021-00507-2 (PMC8783477; doi:10.1186/s13047-021-00507-2)
Supplement: Supplementary file 1 — Additional file 1. [file 13047_2021_507_MOESM1_ESM.docx]

**Additional file 1** Database search terms

Medline (768) and Embase (1046)

1 exp "FASCIITIS, PLANTAR"/

2 exp "HEEL SPUR"/

3 ((PLANTAR OR HEEL OR CALCANE*) ADJ2 PAIN*).ti,ab

4 ((baxter* OR plantar OR calcaneal OR heel) ADJ2 neuropath*).ti,ab

5 exp "DIAGNOSTIC IMAGING"/

6 (magnetic resonance imaging OR MR OR MRI).ti,ab

7 (radiograph* OR x-ray* OR roentgen*).ti,ab

8 (CT OR compute* tomography* OR tomography*).ti,ab

9 (ultrasound* OR ultrasonograph* OR echograph* OR sonograph* OR elastograph*).ti,ab

10 (scintigraph* OR scintigram* OR scintiscan*).ti,ab

11 ((bone* OR radionuclide OR radioisotope) ADJ2 (scan* OR imag*)).ti,ab

12 (1 OR 2 OR 3 OR 4 OR 5)

13 (6 OR 7 OR 8 OR 9 OR 10 OR 11)

14 (12 AND 13)

CINAHL (561)

1 (MM "Plantar Fasciitis")

2 (MM "Heel Spur")

3 (PLANTAR OR HEEL OR CALCANE*) N2 PAIN*

4 (baxter* OR plantar OR calcaneal OR heel) N2 neuropath*

5 (MH "Diagnostic Imaging+")

6 (magnetic resonance imaging OR MR OR MRI)

7 (radiograph* OR x-ray* OR roentgen*)

8 (CT scan OR computed tomograph* OR tomograph*)

9 (ultrasound* OR ultrasonograph* OR echograph* OR sonograph* OR elastograph*)

10 ((bone* OR radionuclide OR radioisotope) N2 (scan* OR imag*))

11 (scintigraph* OR scintigram* OR scintiscan*)

12 S1 OR S2 OR S3 OR S4

13 S5 OR S6 OR S7 OR S8 OR S9 OR S10 OR S11

14 12 AND 13

Cochrane (362)

1. MeSH Fasciitis, Plantar exp

2. MeSH Heel Spur exp

3. (plantar fasci*)

4. (plantar or heel or calcane*) near pain*

5. (baxter* OR plantar OR calcaneal OR heel) near neuropath*

6. MeSH Diagnostic imaging

7 (magnetic resonance imaging OR MR OR MRI)

8 (radiograph* OR x-ray* OR roentgen*)

9 (CT scan OR computed tomograph* OR tomograph*)

10 (ultrasound* OR ultrasonograph* OR echograph* OR sonograph* OR elastograph*)

11 ((bone* OR radionuclide OR radioisotope) near (scan* OR imag*))

12 (scintigraph* OR scintigram* OR scintiscan*)

13 1 or 2 or 3 or 4 or 5

14 6 or 7 or 8 or 9 or 10 or 11 or 12

15 13 AND 14

Sportsdiscus (382)

1 (DE Plantar fasciitis)

2 (DE Heel bone)

3 (PLANTAR OR HEEL OR CALCANE*) N2 PAIN*

4 (baxter* OR plantar OR calcaneal OR heel) N2 neuropath*

5 (DE diagnostic imaging) exp

6 (magnetic resonance imaging OR MR OR MRI)

7 (radiograph* OR x-ray* OR roentgen*)

8 (CT scan OR computed tomograph* OR tomograph*)

9 (ultrasound* OR ultrasonograph* OR echograph* OR sonograph* OR elastograph*)

10 ((bone* OR radionuclide OR radioisotope) N2 (scan* OR imag*))

11 (scintigraph* OR scintigram* OR scintiscan*)

12 S1 OR S2 OR S3 OR S4

13 S5 OR S6 OR S7 OR S8 OR S9 OR S10 OR S11

14 12 AND 13
