# Supplementary material for: Medical imaging for plantar heel pain: a systematic review and meta-analysis
Source: J Foot Ankle Res. 2022 Jan 22;15:4. doi: 10.1186/s13047-021-00507-2 (PMC8783477; doi:10.1186/s13047-021-00507-2)
Supplement: Supplementary file 2 — Additional file 2. [file 13047_2021_507_MOESM2_ESM.docx]

**Additional file 2** List of excluded articles and reasons

| **Authors (date)** | **Imaging modality** | **Grounds for exclusion** |
| --- | --- | --- |
| Abdel-Wahab et al (2008) | Ultrasound/ MRI | Did not exclude participants with endocrine conditions. |
| Amis et al (1988) | Radiograph | No comparison to an asymptomatic control group. |
| Chen et al (2012) | Ultrasound | Unable to access comparative data. |
| Gamba et al (2018) | Ultrasound/MRI | No comparison to an asymptomatic control group. |
| Granado et al (2019) | Ultrasound | Similar data reported in another article (Granado et al 2018). |
| Hall et all (1996) | MRI | Did not exclude participants with endocrine conditions. |
| Johal & Milner | Radiograph | Unable to ascertain whether control group was asymptomatic of PHP. |
| Li et al (2018) | Ultrasound | Non-English language. |
| O’Duffy etal (1998) | Scintigraphy | Did not exclude participants with endocrine conditions. |
| Ribeiro et al (2011) | Digital photography | Not a medical imaging study. |
| Vohra et al (2002) | Ultrasound | Comparison of asymptomatic and symptomatic bands of the plantar fascia in participants with PHP, rather than comparison to an asymptomatic control group. |
| Wainwright et al (1995) | Radiograph | Unable to ascertain whether control group was asymptomatic of PHP. |
| Wearing et al (2004) | Ultrasound | Similar data reported in another article (Wearing et al 2007) . |
| Williams et al (1987) | Radiograph/ Scintigraphy | Radiograph data excluded only. Unable to ascertain whether radiograph control group was asymptomatic of PHP. Scintigraphy data was included. |
| Zhang et al (2014) | 2014 | Non-English language. |
