# Supplementary material for: Medical imaging for plantar heel pain: a systematic review and meta-analysis
Source: J Foot Ankle Res. 2022 Jan 22;15:4. doi: 10.1186/s13047-021-00507-2 (PMC8783477; doi:10.1186/s13047-021-00507-2)
Supplement: Supplementary file 3 — Additional file 3. [file 13047_2021_507_MOESM3_ESM.docx]

**Additional file 3** NIH Quality Assessment Tool for observational cohort and cross-sectional studies

| Study: **Aggarwal et al (2020)** |
| --- |

| Criteria | Yes | No | Other (CD, NR, NA)* |
| --- | --- | --- | --- |
| 1. Was the research question or objective in this paper clearly stated? | * |  |  |
| 2. Was the study population clearly specified and defined? | * |  |  |
| 3. Was the participation rate of eligible persons at least 50%? |  |  | NR |
| 4. Were all the subjects selected or recruited from the same or similar populations (including the same time period)? Were inclusion and exclusion criteria for being in the study prespecified and applied uniformly to all participants? | * |  |  |
| 5. Was a sample size justification, power description, or variance and effect estimates provided? |  | * |  |
| 6. For the analyses in this paper, were the exposure(s) of interest measured prior to the outcome(s) being measured? |  | * | Cross-sectional study |
| 7. Was the timeframe sufficient so that one could reasonably expect to see an association between exposure and outcome if it existed? |  | * | Cross-sectional study |
| 8. For exposures that can vary in amount or level, did the study examine different levels of the exposure as related to the outcome (e.g., categories of exposure, or exposure measured as continuous variable)? | * |  |  |
| 9. Were the exposure measures (independent variables) clearly defined, valid, reliable, and implemented consistently across all study participants? | * |  |  |
| 10. Was the exposure(s) assessed more than once over time? |  | * | Cross-sectional study |
| 11. Were the outcome measures (dependent variables) clearly defined, valid, reliable, and implemented consistently across all study participants? |  | * | PHP severity not measured |
| 12. Were the outcome assessors blinded to the exposure status of participants? |  | NR |  |
| 13. Was loss to follow-up after baseline 20% or less? |  | * | Cross-sectional study |
| 14. Were key potential confounding variables measured and adjusted statistically for their impact on the relationship between exposure(s) and outcome(s) |  | * | Age matched but no statistical adjustment for potential confounding variables. |
|  |  |  |  |

| **Quality Rating (Good, Fair, or Poor)** |
| --- |
| Rater #1 initials: CD: Poor |
| Rater #2 initials: MRK: Poor |
| Additional Comments (If POOR, please state why):  No blinding of assessors, no statistical adjustment for potential confounding variables. |

*CD, cannot determine; NA, not applicable; NR, not reported

NIH Quality Assessment Tool for observational cohort and cross-sectional

| Study: **Akfirat et al (2003)** |
| --- |

| Criteria | Yes | No | Other (CD, NR, NA)* |
| --- | --- | --- | --- |
| 1. Was the research question or objective in this paper clearly stated? |  | * | Objective not clear |
| 2. Was the study population clearly specified and defined? | * |  |  |
| 3. Was the participation rate of eligible persons at least 50%? |  |  | NR |
| 4. Were all the subjects selected or recruited from the same or similar populations (including the same time period)? Were inclusion and exclusion criteria for being in the study prespecified and applied uniformly to all participants? |  | * | Source and selection of participants unclear  Recruitment time period not mentioned  Inclusion/exclusion criteria unclear |
| 5. Was a sample size justification, power description, or variance and effect estimates provided? |  | * |  |
| 6. For the analyses in this paper, were the exposure(s) of interest measured prior to the outcome(s) being measured? |  | * | Cross-sectional study |
| 7. Was the timeframe sufficient so that one could reasonably expect to see an association between exposure and outcome if it existed? |  | * | Cross-sectional study |
| 8. For exposures that can vary in amount or level, did the study examine different levels of the exposure as related to the outcome (e.g., categories of exposure, or exposure measured as continuous variable)? | * |  |  |
| 9. Were the exposure measures (independent variables) clearly defined, valid, reliable, and implemented consistently across all study participants? | * |  |  |
| 10. Was the exposure(s) assessed more than once over time? |  | * | Cross-sectional study |
| 11. Were the outcome measures (dependent variables) clearly defined, valid, reliable, and implemented consistently across all study participants? |  | * | No PHP severity reported |
| 12. Were the outcome assessors blinded to the exposure status of participants? |  | * |  |
| 13. Was loss to follow-up after baseline 20% or less? |  | * | Cross-sectional study |
| 14. Were key potential confounding variables measured and adjusted statistically for their impact on the relationship between exposure(s) and outcome(s) | * |  | Similar characteristics between groups |
|  |  |  |  |

| **Quality Rating (Good, Fair, or Poor)** |
| --- |
| Rater #1 initials: CD: Poor |
| Rater #2 initials: MRK: Poor |
| Additional Comments (If POOR, please state why):  Research question unclear, source and selection of participants unclear, inclusion/exclusion criteria unclear, no blinding of assessors. |

*CD, cannot determine; NA, not applicable; NR, not reported

NIH Quality Assessment Tool for observational cohort and cross-sectional

| Study: **Berkowitz et al (1991)** |
| --- |

| Criteria | Yes | No | Other (CD, NR, NA)* |
| --- | --- | --- | --- |
| 1. Was the research question or objective in this paper clearly stated? | * |  |  |
| 2. Was the study population clearly specified and defined? | * |  |  |
| 3. Was the participation rate of eligible persons at least 50%? |  |  | NR |
| 4. Were all the subjects selected or recruited from the same or similar populations (including the same time period)? Were inclusion and exclusion criteria for being in the study prespecified and applied uniformly to all participants? |  | * | Inclusion & exclusion criteria unclear  Source of participants unclear |
| 5. Was a sample size justification, power description, or variance and effect estimates provided? |  | * |  |
| 6. For the analyses in this paper, were the exposure(s) of interest measured prior to the outcome(s) being measured? |  | * | Cross-sectional study |
| 7. Was the timeframe sufficient so that one could reasonably expect to see an association between exposure and outcome if it existed? |  | * | Cross-sectional study |
| 8. For exposures that can vary in amount or level, did the study examine different levels of the exposure as related to the outcome (e.g., categories of exposure, or exposure measured as continuous variable)? | * |  |  |
| 9. Were the exposure measures (independent variables) clearly defined, valid, reliable, and implemented consistently across all study participants? | * |  |  |
| 10. Was the exposure(s) assessed more than once over time? |  | * | Cross-sectional study |
| 11. Were the outcome measures (dependent variables) clearly defined, valid, reliable, and implemented consistently across all study participants? |  | * | No PHP severity reported |
| 12. Were the outcome assessors blinded to the exposure status of participants? |  |  | NR |
| 13. Was loss to follow-up after baseline 20% or less? |  | * | Cross-sectional study |
| 14. Were key potential confounding variables measured and adjusted statistically for their impact on the relationship between exposure(s) and outcome(s) |  | * | NR – limited mention of statistical analysis |
|  |  |  |  |

| **Quality Rating (Good, Fair, or Poor)** |
| --- |
| Rater #1 initials: CD: Poor |
| Rater #2 initials: MK: Poor |
| Additional Comments (If POOR, please state why):  Source and selection of all participants unclear, inclusion/exclusion criteria unclear, no reported blinding of assessors, limited information regarding statistical analysis methods or results. |

*CD, cannot determine; NA, not applicable; NR, not reported

NIH Quality Assessment Tool for observational cohort and cross-sectional

| Study: **Bygrave et al (1998)** |
| --- |

| Criteria | Yes | No | Other (CD, NR, NA)* |
| --- | --- | --- | --- |
| 1. Was the research question or objective in this paper clearly stated? | * |  |  |
| 2. Was the study population clearly specified and defined? | * |  |  |
| 3. Was the participation rate of eligible persons at least 50%? |  |  | NR |
| 4. Were all the subjects selected or recruited from the same or similar populations (including the same time period)? Were inclusion and exclusion criteria for being in the study prespecified and applied uniformly to all participants? |  | * | Source and selection of participants unclear  No recruitment time period mentioned |
| 5. Was a sample size justification, power description, or variance and effect estimates provided? |  | * |  |
| 6. For the analyses in this paper, were the exposure(s) of interest measured prior to the outcome(s) being measured? |  | * | Cross-sectional study |
| 7. Was the timeframe sufficient so that one could reasonably expect to see an association between exposure and outcome if it existed? |  | * | Cross-sectional study |
| 8. For exposures that can vary in amount or level, did the study examine different levels of the exposure as related to the outcome (e.g., categories of exposure, or exposure measured as continuous variable)? | * |  |  |
| 9. Were the exposure measures (independent variables) clearly defined, valid, reliable, and implemented consistently across all study participants? | * |  |  |
| 10. Was the exposure(s) assessed more than once over time? |  | * | Cross-sectional study |
| 11. Were the outcome measures (dependent variables) clearly defined, valid, reliable, and implemented consistently across all study participants? |  | * | No PHP severity reported |
| 12. Were the outcome assessors blinded to the exposure status of participants? |  |  | NR |
| 13. Was loss to follow-up after baseline 20% or less? |  | * | Cross-sectional study |
| 14. Were key potential confounding variables measured and adjusted statistically for their impact on the relationship between exposure(s) and outcome(s) |  | * |  |
|  |  |  |  |

| **Quality Rating (Good, Fair, or Poor)** |
| --- |
| Rater #1 initials: CD: Poor |
| Rater #2 initials: MRK: Poor |
| Additional Comments (If POOR, please state why):  Source and selection of participants unclear, no PHP severity reported, no blinding of assessors, no adjustment for confounding variables. |

*CD, cannot determine; NA, not applicable; NR, not reported

NIH Quality Assessment Tool for observational cohort and cross-sectional

| Study: **Cardinal et al (1996)** |
| --- |

| Criteria | Yes | No | Other (CD, NR, NA)* |
| --- | --- | --- | --- |
| 1. Was the research question or objective in this paper clearly stated? | * |  |  |
| 2. Was the study population clearly specified and defined? | * |  |  |
| 3. Was the participation rate of eligible persons at least 50%? |  |  | NR |
| 4. Were all the subjects selected or recruited from the same or similar populations (including the same time period)? Were inclusion and exclusion criteria for being in the study prespecified and applied uniformly to all participants? |  | * | Source and selection of participants unclear  No recruitment time period mentioned  Inclusion/exclusion criteria unclear |
| 5. Was a sample size justification, power description, or variance and effect estimates provided? |  | * |  |
| 6. For the analyses in this paper, were the exposure(s) of interest measured prior to the outcome(s) being measured? |  | * | Cross-sectional study |
| 7. Was the timeframe sufficient so that one could reasonably expect to see an association between exposure and outcome if it existed? |  | * | Cross-sectional study |
| 8. For exposures that can vary in amount or level, did the study examine different levels of the exposure as related to the outcome (e.g., categories of exposure, or exposure measured as continuous variable)? | * |  |  |
| 9. Were the exposure measures (independent variables) clearly defined, valid, reliable, and implemented consistently across all study participants? |  | * | Probe placement not standardised |
| 10. Was the exposure(s) assessed more than once over time? |  | * | Cross-sectional study |
| 11. Were the outcome measures (dependent variables) clearly defined, valid, reliable, and implemented consistently across all study participants? |  | * | PHP severity not measured |
| 12. Were the outcome assessors blinded to the exposure status of participants? |  | * |  |
| 13. Was loss to follow-up after baseline 20% or less? |  | * | Cross-sectional study |
| 14. Were key potential confounding variables measured and adjusted statistically for their impact on the relationship between exposure(s) and outcome(s) |  | * |  |
|  |  |  |  |

| **Quality Rating (Good, Fair, or Poor)** |
| --- |
| Rater #1 initials: CD: Poor |
| Rater #2 initials: MRK: Poor |
| Additional Comments (If POOR, please state why):  Source and selection of participants unclear, inclusion/exclusion criteria, probe placement not standardised, no blinding of assessors, limited information regarding statistical analysis methods or results. |

*CD, cannot determine; NA, not applicable; NR, not reported

NIH Quality Assessment Tool for observational cohort and cross-sectional

| Study: **Cetin et al (2001)** |
| --- |

| Criteria | Yes | No | Other (CD, NR, NA)* |
| --- | --- | --- | --- |
| 1. Was the research question or objective in this paper clearly stated? | * |  |  |
| 2. Was the study population clearly specified and defined? | * |  |  |
| 3. Was the participation rate of eligible persons at least 50%? |  |  | NR |
| 4. Were all the subjects selected or recruited from the same or similar populations (including the same time period)? Were inclusion and exclusion criteria for being in the study prespecified and applied uniformly to all participants? |  | * | Source and selection of participants unclear  No recruitment time period mentioned |
| 5. Was a sample size justification, power description, or variance and effect estimates provided? |  | * |  |
| 6. For the analyses in this paper, were the exposure(s) of interest measured prior to the outcome(s) being measured? |  | * | Cross-sectional study |
| 7. Was the timeframe sufficient so that one could reasonably expect to see an association between exposure and outcome if it existed? |  | * | Cross-sectional study |
| 8. For exposures that can vary in amount or level, did the study examine different levels of the exposure as related to the outcome (e.g., categories of exposure, or exposure measured as continuous variable)? | * |  |  |
| 9. Were the exposure measures (independent variables) clearly defined, valid, reliable, and implemented consistently across all study participants? | * |  |  |
| 10. Was the exposure(s) assessed more than once over time? |  | * | Cross-sectional study |
| 11. Were the outcome measures (dependent variables) clearly defined, valid, reliable, and implemented consistently across all study participants? | * |  |  |
| 12. Were the outcome assessors blinded to the exposure status of participants? |  |  | NR |
| 13. Was loss to follow-up after baseline 20% or less? |  | * | Cross-sectional study |
| 14. Were key potential confounding variables measured and adjusted statistically for their impact on the relationship between exposure(s) and outcome(s) | * |  | Similar characteristics between groups |
|  |  |  |  |

| **Quality Rating (Good, Fair, or Poor)** |
| --- |
| Rater #1 initials: CD: Fair |
| Rater #2 initials: MRK: Fair |
| Additional Comments (If POOR, please state why): |

*CD, cannot determine; NA, not applicable; NR, not reported

NIH Quality Assessment Tool for observational cohort and cross-sectional

| Study: **Chen et al (2013)** |
| --- |

| Criteria | Yes | No | Other (CD, NR, NA)* |
| --- | --- | --- | --- |
| 1. Was the research question or objective in this paper clearly stated? | * |  |  |
| 2. Was the study population clearly specified and defined? | * |  |  |
| 3. Was the participation rate of eligible persons at least 50%? |  |  | NR |
| 4. Were all the subjects selected or recruited from the same or similar populations (including the same time period)? Were inclusion and exclusion criteria for being in the study prespecified and applied uniformly to all participants? | * |  |  |
| 5. Was a sample size justification, power description, or variance and effect estimates provided? |  | * |  |
| 6. For the analyses in this paper, were the exposure(s) of interest measured prior to the outcome(s) being measured? |  | * | Cross-sectional study |
| 7. Was the timeframe sufficient so that one could reasonably expect to see an association between exposure and outcome if it existed? |  | * | Cross-sectional study |
| 8. For exposures that can vary in amount or level, did the study examine different levels of the exposure as related to the outcome (e.g., categories of exposure, or exposure measured as continuous variable)? | * |  |  |
| 9. Were the exposure measures (independent variables) clearly defined, valid, reliable, and implemented consistently across all study participants? | * |  |  |
| 10. Was the exposure(s) assessed more than once over time? |  | * | Cross-sectional study |
| 11. Were the outcome measures (dependent variables) clearly defined, valid, reliable, and implemented consistently across all study participants? | * |  |  |
| 12. Were the outcome assessors blinded to the exposure status of participants? |  |  | NR |
| 13. Was loss to follow-up after baseline 20% or less? |  | * | Cross-sectional study |
| 14. Were key potential confounding variables measured and adjusted statistically for their impact on the relationship between exposure(s) and outcome(s) | * |  |  |
|  |  |  |  |

| **Quality Rating (Good, Fair, or Poor)** |
| --- |
| Rater #1 initials: CD: Fair |
| Rater #2 initials: MRK: Fair |
| Additional Comments (If POOR, please state why): |

*CD, cannot determine; NA, not applicable; NR, not reported

NIH Quality Assessment Tool for observational cohort and cross-sectional

| Study: **Cheng et al (2012)** |
| --- |

| Criteria | Yes | No | Other (CD, NR, NA)* |
| --- | --- | --- | --- |
| 1. Was the research question or objective in this paper clearly stated? | * |  |  |
| 2. Was the study population clearly specified and defined? | * |  |  |
| 3. Was the participation rate of eligible persons at least 50%? |  |  | NR |
| 4. Were all the subjects selected or recruited from the same or similar populations (including the same time period)? Were inclusion and exclusion criteria for being in the study prespecified and applied uniformly to all participants? |  | * | Source and selection of participants unclear  No recruitment time period mentioned  Inclusion/exclusion criteria unclear |
| 5. Was a sample size justification, power description, or variance and effect estimates provided? |  | * |  |
| 6. For the analyses in this paper, were the exposure(s) of interest measured prior to the outcome(s) being measured? |  | * | Cross-sectional study |
| 7. Was the timeframe sufficient so that one could reasonably expect to see an association between exposure and outcome if it existed? |  | * | Cross-sectional study |
| 8. For exposures that can vary in amount or level, did the study examine different levels of the exposure as related to the outcome (e.g., categories of exposure, or exposure measured as continuous variable)? | * |  |  |
| 9. Were the exposure measures (independent variables) clearly defined, valid, reliable, and implemented consistently across all study participants? |  | * | Probe placement not standardised |
| 10. Was the exposure(s) assessed more than once over time? |  | * | Cross-sectional study |
| 11. Were the outcome measures (dependent variables) clearly defined, valid, reliable, and implemented consistently across all study participants? |  | * | PHP severity not measured |
| 12. Were the outcome assessors blinded to the exposure status of participants? | * |  |  |
| 13. Was loss to follow-up after baseline 20% or less? |  | * | Cross-sectional study |
| 14. Were key potential confounding variables measured and adjusted statistically for their impact on the relationship between exposure(s) and outcome(s) |  | * |  |
|  |  |  |  |

| **Quality Rating (Good, Fair, or Poor)** |
| --- |
| Rater #1 initials: CD: Poor |
| Rater #2 initials: MRK: Poor |
| Additional Comments (If POOR, please state why):  Source and selection of participants unclear, inclusion/exclusion criteria unclear, probe placement not standardised, PHP severity not measured, potential confounding variables not reported. |

*CD, cannot determine; NA, not applicable; NR, not reported

NIH Quality Assessment Tool for observational cohort and cross-sectional

| Study: **Cheung et al (2016)** |
| --- |

| Criteria | Yes | No | Other (CD, NR, NA)* |
| --- | --- | --- | --- |
| 1. Was the research question or objective in this paper clearly stated? | * |  |  |
| 2. Was the study population clearly specified and defined? | * |  |  |
| 3. Was the participation rate of eligible persons at least 50%? |  |  | NR |
| 4. Were all the subjects selected or recruited from the same or similar populations (including the same time period)? Were inclusion and exclusion criteria for being in the study prespecified and applied uniformly to all participants? |  | * | Source and selection of participants unclear  No recruitment time period mentioned  Inclusion/exclusion criteria unclear |
| 5. Was a sample size justification, power description, or variance and effect estimates provided? |  | * |  |
| 6. For the analyses in this paper, were the exposure(s) of interest measured prior to the outcome(s) being measured? |  | * | Cross-sectional study |
| 7. Was the timeframe sufficient so that one could reasonably expect to see an association between exposure and outcome if it existed? |  | * | Cross-sectional study |
| 8. For exposures that can vary in amount or level, did the study examine different levels of the exposure as related to the outcome (e.g., categories of exposure, or exposure measured as continuous variable)? | * |  |  |
| 9. Were the exposure measures (independent variables) clearly defined, valid, reliable, and implemented consistently across all study participants? | * |  |  |
| 10. Was the exposure(s) assessed more than once over time? |  | * | Cross-sectional study |
| 11. Were the outcome measures (dependent variables) clearly defined, valid, reliable, and implemented consistently across all study participants? | * |  |  |
| 12. Were the outcome assessors blinded to the exposure status of participants? |  |  | NR |
| 13. Was loss to follow-up after baseline 20% or less? |  | * | Cross-sectional study |
| 14. Were key potential confounding variables measured and adjusted statistically for their impact on the relationship between exposure(s) and outcome(s) | * |  | Similar characteristics between groups |
|  |  |  |  |

| **Quality Rating (Good, Fair, or Poor)** |
| --- |
| Rater #1 initials:CD: Fair |
| Rater #2 initials: MRK: Fair |
| Additional Comments (If POOR, please state why): |

*CD, cannot determine; NA, not applicable; NR, not reported

NIH Quality Assessment Tool for observational cohort and cross-sectional

| Study: **Fabrikant et al (2011)** |
| --- |

| Criteria | Yes | No | Other (CD, NR, NA)* |
| --- | --- | --- | --- |
| 1. Was the research question or objective in this paper clearly stated? | * |  |  |
| 2. Was the study population clearly specified and defined? | * |  |  |
| 3. Was the participation rate of eligible persons at least 50%? |  |  | NR |
| 4. Were all the subjects selected or recruited from the same or similar populations (including the same time period)? Were inclusion and exclusion criteria for being in the study prespecified and applied uniformly to all participants? | * |  | Recruitment period not detailed. |
| 5. Was a sample size justification, power description, or variance and effect estimates provided? |  | * |  |
| 6. For the analyses in this paper, were the exposure(s) of interest measured prior to the outcome(s) being measured? |  | * | Cross-sectional study |
| 7. Was the timeframe sufficient so that one could reasonably expect to see an association between exposure and outcome if it existed? |  | * | Cross-sectional study |
| 8. For exposures that can vary in amount or level, did the study examine different levels of the exposure as related to the outcome (e.g., categories of exposure, or exposure measured as continuous variable)? | * |  |  |
| 9. Were the exposure measures (independent variables) clearly defined, valid, reliable, and implemented consistently across all study participants? | * |  |  |
| 10. Was the exposure(s) assessed more than once over time? |  | * | Cross-sectional study |
| 11. Were the outcome measures (dependent variables) clearly defined, valid, reliable, and implemented consistently across all study participants? | * |  |  |
| 12. Were the outcome assessors blinded to the exposure status of participants? |  | NR |  |
| 13. Was loss to follow-up after baseline 20% or less? |  | * | Cross-sectional study |
| 14. Were key potential confounding variables measured and adjusted statistically for their impact on the relationship between exposure(s) and outcome(s) | * |  |  |
|  |  |  |  |

| **Quality Rating (Good, Fair, or Poor)** |
| --- |
| Rater #1 initials: CD: Fair |
| Rater #2 initials: MRK: Fair |
| Additional Comments (If POOR, please state why): |

*CD, cannot determine; NA, not applicable; NR, not reported

NIH Quality Assessment Tool for observational cohort and cross-sectional

| Study: **Fernandez-Lao et al (2016)** |
| --- |

| Criteria | Yes | No | Other (CD, NR, NA)* |
| --- | --- | --- | --- |
| 1. Was the research question or objective in this paper clearly stated? | * |  |  |
| 2. Was the study population clearly specified and defined? | * |  |  |
| 3. Was the participation rate of eligible persons at least 50%? |  |  | NR |
| 4. Were all the subjects selected or recruited from the same or similar populations (including the same time period)? Were inclusion and exclusion criteria for being in the study prespecified and applied uniformly to all participants? | * |  |  |
| 5. Was a sample size justification, power description, or variance and effect estimates provided? | * |  |  |
| 6. For the analyses in this paper, were the exposure(s) of interest measured prior to the outcome(s) being measured? |  | * | Cross-sectional study |
| 7. Was the timeframe sufficient so that one could reasonably expect to see an association between exposure and outcome if it existed? |  | * | Cross-sectional study |
| 8. For exposures that can vary in amount or level, did the study examine different levels of the exposure as related to the outcome (e.g., categories of exposure, or exposure measured as continuous variable)? | * |  |  |
| 9. Were the exposure measures (independent variables) clearly defined, valid, reliable, and implemented consistently across all study participants? |  | * | Probe placement not standardised |
| 10. Was the exposure(s) assessed more than once over time? |  | * | Cross-sectional study |
| 11. Were the outcome measures (dependent variables) clearly defined, valid, reliable, and implemented consistently across all study participants? | * |  |  |
| 12. Were the outcome assessors blinded to the exposure status of participants? |  |  | NR |
| 13. Was loss to follow-up after baseline 20% or less? |  | * | Cross-sectional study |
| 14. Were key potential confounding variables measured and adjusted statistically for their impact on the relationship between exposure(s) and outcome(s) | * |  |  |
|  |  |  |  |

| **Quality Rating (Good, Fair, or Poor)** |
| --- |
| Rater #1 initials: CD: Fair |
| Rater #2 initials: MRK: Fair |
| Additional Comments (If POOR, please state why): |

*CD, cannot determine; NA, not applicable; NR, not reported

NIH Quality Assessment Tool for observational cohort and cross-sectional

Study: **Finkenstaedt et al (2018)**

| Criteria | Yes | No | Other (CD, NR, NA)* |
| --- | --- | --- | --- |
| 1. Was the research question or objective in this paper clearly stated? | * |  |  |
| 2. Was the study population clearly specified and defined? | * |  |  |
| 3. Was the participation rate of eligible persons at least 50%? |  |  | NR |
| 4. Were all the subjects selected or recruited from the same or similar populations (including the same time period)? Were inclusion and exclusion criteria for being in the study prespecified and applied uniformly to all participants? |  | * | Heterogeneity in control group.  No recruitment time period mentioned |
| 5. Was a sample size justification, power description, or variance and effect estimates provided? |  | * |  |
| 6. For the analyses in this paper, were the exposure(s) of interest measured prior to the outcome(s) being measured? |  | * | Retrospective study |
| 7. Was the timeframe sufficient so that one could reasonably expect to see an association between exposure and outcome if it existed? |  | * | Retrospective study |
| 8. For exposures that can vary in amount or level, did the study examine different levels of the exposure as related to the outcome (e.g., categories of exposure, or exposure measured as continuous variable)? | * |  |  |
| 9. Were the exposure measures (independent variables) clearly defined, valid, reliable, and implemented consistently across all study participants? | * |  |  |
| 10. Was the exposure(s) assessed more than once over time? |  | * | Cross-sectional study |
| 11. Were the outcome measures (dependent variables) clearly defined, valid, reliable, and implemented consistently across all study participants? |  | * | PHP severity not measured |
| 12. Were the outcome assessors blinded to the exposure status of participants? | * |  |  |
| 13. Was loss to follow-up after baseline 20% or less? |  | * | Retrospective study |
| 14. Were key potential confounding variables measured and adjusted statistically for their impact on the relationship between exposure(s) and outcome(s) | * |  |  |
|  |  |  |  |

| **Quality Rating (Good, Fair, or Poor)** |
| --- |
| Rater #1 initials: CD: Fair |
| Rater #2 initials: MRK: Fair |
| Additional Comments (If POOR, please state why): |

*CD, cannot determine; NA, not applicable; NR, not reported

NIH Quality Assessment Tool for observational cohort and cross-sectional

| Study: **Gatz et al (2020)** |
| --- |

| Criteria | Yes | No | Other (CD, NR, NA)* |
| --- | --- | --- | --- |
| 1. Was the research question or objective in this paper clearly stated? | * |  |  |
| 2. Was the study population clearly specified and defined? | * |  |  |
| 3. Was the participation rate of eligible persons at least 50%? |  |  | NR |
| 4. Were all the subjects selected or recruited from the same or similar populations (including the same time period)? Were inclusion and exclusion criteria for being in the study prespecified and applied uniformly to all participants? | * |  |  |
| 5. Was a sample size justification, power description, or variance and effect estimates provided? |  | * |  |
| 6. For the analyses in this paper, were the exposure(s) of interest measured prior to the outcome(s) being measured? |  | * | Cross-sectional study |
| 7. Was the timeframe sufficient so that one could reasonably expect to see an association between exposure and outcome if it existed? |  | * | Cross-sectional study |
| 8. For exposures that can vary in amount or level, did the study examine different levels of the exposure as related to the outcome (e.g., categories of exposure, or exposure measured as continuous variable)? | * |  |  |
| 9. Were the exposure measures (independent variables) clearly defined, valid, reliable, and implemented consistently across all study participants? | * |  |  |
| 10. Was the exposure(s) assessed more than once over time? |  | * | Cross-sectional study |
| 11. Were the outcome measures (dependent variables) clearly defined, valid, reliable, and implemented consistently across all study participants? | * |  |  |
| 12. Were the outcome assessors blinded to the exposure status of participants? | * |  |  |
| 13. Was loss to follow-up after baseline 20% or less? |  | * | Cross-sectional study |
| 14. Were key potential confounding variables measured and adjusted statistically for their impact on the relationship between exposure(s) and outcome(s) | * |  |  |
|  |  |  |  |

| **Quality Rating (Good, Fair, or Poor)** |
| --- |
| Rater #1 initials: CD: Good |
| Rater #2 initials: MRK: Good |
| Additional Comments (If POOR, please state why): |

*CD, cannot determine; NA, not applicable; NR, not reported

NIH Quality Assessment Tool for observational cohort and cross-sectional

| Study: **Genc et al (2005)** |
| --- |

| Criteria | Yes | No | Other (CD, NR, NA)* |
| --- | --- | --- | --- |
| 1. Was the research question or objective in this paper clearly stated? | * |  |  |
| 2. Was the study population clearly specified and defined? | * |  |  |
| 3. Was the participation rate of eligible persons at least 50%? |  |  | NR |
| 4. Were all the subjects selected or recruited from the same or similar populations (including the same time period)? Were inclusion and exclusion criteria for being in the study prespecified and applied uniformly to all participants? |  | * | Source and selection of participants unclear  No recruitment time period mentioned |
| 5. Was a sample size justification, power description, or variance and effect estimates provided? |  | * |  |
| 6. For the analyses in this paper, were the exposure(s) of interest measured prior to the outcome(s) being measured? |  | * | Cross-sectional study |
| 7. Was the timeframe sufficient so that one could reasonably expect to see an association between exposure and outcome if it existed? |  | * | Cross-sectional study |
| 8. For exposures that can vary in amount or level, did the study examine different levels of the exposure as related to the outcome (e.g., categories of exposure, or exposure measured as continuous variable)? | * |  |  |
| 9. Were the exposure measures (independent variables) clearly defined, valid, reliable, and implemented consistently across all study participants? | * |  |  |
| 10. Was the exposure(s) assessed more than once over time? |  | * | Cross-sectional study |
| 11. Were the outcome measures (dependent variables) clearly defined, valid, reliable, and implemented consistently across all study participants? | * |  |  |
| 12. Were the outcome assessors blinded to the exposure status of participants? |  | * |  |
| 13. Was loss to follow-up after baseline 20% or less? |  | * | Cross-sectional study |
| 14. Were key potential confounding variables measured and adjusted statistically for their impact on the relationship between exposure(s) and outcome(s) | * |  | Matched controls (age, gender, BMI) |
|  |  |  |  |

| **Quality Rating (Good, Fair, or Poor)** |
| --- |
| Rater #1 initials: CD: Fair |
| Rater #2 initials: MRK: Fair |
| Additional Comments (If POOR, please state why): |

*CD, cannot determine; NA, not applicable; NR, not reported

NIH Quality Assessment Tool for observational cohort and cross-sectional

| Study: **Gibbon et al (1999)** |
| --- |

| Criteria | Yes | No | Other (CD, NR, NA)* |
| --- | --- | --- | --- |
| 1. Was the research question or objective in this paper clearly stated? | * |  |  |
| 2. Was the study population clearly specified and defined? | * |  |  |
| 3. Was the participation rate of eligible persons at least 50%? |  |  | NR |
| 4. Were all the subjects selected or recruited from the same or similar populations (including the same time period)? Were inclusion and exclusion criteria for being in the study prespecified and applied uniformly to all participants? |  | * | Inclusion/exclusion criteria unclear |
| 5. Was a sample size justification, power description, or variance and effect estimates provided? |  | * |  |
| 6. For the analyses in this paper, were the exposure(s) of interest measured prior to the outcome(s) being measured? |  | * | Cross-sectional study |
| 7. Was the timeframe sufficient so that one could reasonably expect to see an association between exposure and outcome if it existed? |  | * | Cross-sectional study |
| 8. For exposures that can vary in amount or level, did the study examine different levels of the exposure as related to the outcome (e.g., categories of exposure, or exposure measured as continuous variable)? | * |  |  |
| 9. Were the exposure measures (independent variables) clearly defined, valid, reliable, and implemented consistently across all study participants? | * |  |  |
| 10. Was the exposure(s) assessed more than once over time? |  | * | Cross-sectional study |
| 11. Were the outcome measures (dependent variables) clearly defined, valid, reliable, and implemented consistently across all study participants? |  | * | PHP severity not measured |
| 12. Were the outcome assessors blinded to the exposure status of participants? |  | * |  |
| 13. Was loss to follow-up after baseline 20% or less? |  | * | Cross-sectional study |
| 14. Were key potential confounding variables measured and adjusted statistically for their impact on the relationship between exposure(s) and outcome(s) |  | * |  |
|  |  |  |  |

| **Quality Rating (Good, Fair, or Poor)** |
| --- |
| Rater #1 initials: CD: Poor |
| Rater #2 initials: MRK: Poor |
| Additional Comments (If POOR, please state why):  Inclusion/exclusion criteria unclear, no blinding of assessors, PHP severity not measured, statistical analysis did not account for potential confounding variables. |

*CD, cannot determine; NA, not applicable; NR, not reported

NIH Quality Assessment Tool for observational cohort and cross-sectional

| Study: **Granado et al (2018)** |
| --- |

| Criteria | Yes | No | Other (CD, NR, NA)* |
| --- | --- | --- | --- |
| 1. Was the research question or objective in this paper clearly stated? | * |  |  |
| 2. Was the study population clearly specified and defined? | * |  |  |
| 3. Was the participation rate of eligible persons at least 50%? |  |  | NR |
| 4. Were all the subjects selected or recruited from the same or similar populations (including the same time period)? Were inclusion and exclusion criteria for being in the study prespecified and applied uniformly to all participants? |  | * | Source and selection of participants unclear  No recruitment time period mentioned |
| 5. Was a sample size justification, power description, or variance and effect estimates provided? |  | * |  |
| 6. For the analyses in this paper, were the exposure(s) of interest measured prior to the outcome(s) being measured? |  | * | Cross-sectional study |
| 7. Was the timeframe sufficient so that one could reasonably expect to see an association between exposure and outcome if it existed? |  | * | Cross-sectional study |
| 8. For exposures that can vary in amount or level, did the study examine different levels of the exposure as related to the outcome (e.g., categories of exposure, or exposure measured as continuous variable)? | * |  |  |
| 9. Were the exposure measures (independent variables) clearly defined, valid, reliable, and implemented consistently across all study participants? | * |  |  |
| 10. Was the exposure(s) assessed more than once over time? |  | * | Cross-sectional study |
| 11. Were the outcome measures (dependent variables) clearly defined, valid, reliable, and implemented consistently across all study participants? |  | * | PHP severity not measured |
| 12. Were the outcome assessors blinded to the exposure status of participants? |  | * |  |
| 13. Was loss to follow-up after baseline 20% or less? |  | * | Cross-sectional study |
| 14. Were key potential confounding variables measured and adjusted statistically for their impact on the relationship between exposure(s) and outcome(s) |  | * |  |
|  |  |  |  |

| **Quality Rating (Good, Fair, or Poor)** |
| --- |
| Rater #1 initials: CD: Poor |
| Rater #2 initials: MRK: Poor |
| Additional Comments (If POOR, please state why):  Source and selection of participants unclear, PHP severity not measured, no blinding of assessors, no adjustment for potential confounding variables. |

*CD, cannot determine; NA, not applicable; NR, not reported

NIH Quality Assessment Tool for observational cohort and cross-sectional

| Study: **Hogan et al (2020)** |
| --- |

| Criteria | Yes | No | Other (CD, NR, NA)* |
| --- | --- | --- | --- |
| 1. Was the research question or objective in this paper clearly stated? | * |  |  |
| 2. Was the study population clearly specified and defined? | * |  |  |
| 3. Was the participation rate of eligible persons at least 50%? |  |  | NR |
| 4. Were all the subjects selected or recruited from the same or similar populations (including the same time period)? Were inclusion and exclusion criteria for being in the study prespecified and applied uniformly to all participants? | * |  |  |
| 5. Was a sample size justification, power description, or variance and effect estimates provided? |  | * |  |
| 6. For the analyses in this paper, were the exposure(s) of interest measured prior to the outcome(s) being measured? |  | * | Cross-sectional study |
| 7. Was the timeframe sufficient so that one could reasonably expect to see an association between exposure and outcome if it existed? |  | * | Cross-sectional study |
| 8. For exposures that can vary in amount or level, did the study examine different levels of the exposure as related to the outcome (e.g., categories of exposure, or exposure measured as continuous variable)? | * |  |  |
| 9. Were the exposure measures (independent variables) clearly defined, valid, reliable, and implemented consistently across all study participants? | * |  |  |
| 10. Was the exposure(s) assessed more than once over time? |  | * | Cross-sectional study |
| 11. Were the outcome measures (dependent variables) clearly defined, valid, reliable, and implemented consistently across all study participants? | * |  |  |
| 12. Were the outcome assessors blinded to the exposure status of participants? |  | * |  |
| 13. Was loss to follow-up after baseline 20% or less? |  | * | Cross-sectional study |
| 14. Were key potential confounding variables measured and adjusted statistically for their impact on the relationship between exposure(s) and outcome(s) | * |  | Similar group characteristics |
|  |  |  |  |

| **Quality Rating (Good, Fair, or Poor)** |
| --- |
| Rater #1 initials: CD: Fair |
| Rater #2 initials: MRK: Fair |
| Additional Comments (If POOR, please state why): |

*CD, cannot determine; NA, not applicable; NR, not reported

NIH Quality Assessment Tool for observational cohort and cross-sectional

| Study: **Kamel et al (2000)** |
| --- |

| Criteria | Yes | No | Other (CD, NR, NA)* |
| --- | --- | --- | --- |
| 1. Was the research question or objective in this paper clearly stated? | * |  |  |
| 2. Was the study population clearly specified and defined? | * |  |  |
| 3. Was the participation rate of eligible persons at least 50%? |  |  | NR |
| 4. Were all the subjects selected or recruited from the same or similar populations (including the same time period)? Were inclusion and exclusion criteria for being in the study prespecified and applied uniformly to all participants? |  | * | Source and selection of participants unclear  No recruitment time period mentioned  Inclusion/exclusion criteria unclear |
| 5. Was a sample size justification, power description, or variance and effect estimates provided? |  | * |  |
| 6. For the analyses in this paper, were the exposure(s) of interest measured prior to the outcome(s) being measured? |  | * | Cross-sectional study |
| 7. Was the timeframe sufficient so that one could reasonably expect to see an association between exposure and outcome if it existed? |  | * | Cross-sectional study |
| 8. For exposures that can vary in amount or level, did the study examine different levels of the exposure as related to the outcome (e.g., categories of exposure, or exposure measured as continuous variable)? | * |  |  |
| 9. Were the exposure measures (independent variables) clearly defined, valid, reliable, and implemented consistently across all study participants? |  | * | Probe placement not standardised |
| 10. Was the exposure(s) assessed more than once over time? |  | * | Cross-sectional study |
| 11. Were the outcome measures (dependent variables) clearly defined, valid, reliable, and implemented consistently across all study participants? |  | * | PHP severity not measured |
| 12. Were the outcome assessors blinded to the exposure status of participants? |  |  | NR |
| 13. Was loss to follow-up after baseline 20% or less? |  | * | Cross-sectional study |
| 14. Were key potential confounding variables measured and adjusted statistically for their impact on the relationship between exposure(s) and outcome(s) |  | * | Matched controls (age, sex), but no description of statistical analysis methods |
|  |  |  |  |

| **Quality Rating (Good, Fair, or Poor)** |
| --- |
| Rater #1 initials: CD: Poor |
| Rater #2 initials: MRK: Poor |
| Additional Comments (If POOR, please state why):  Source and selection of participants unclear, inclusion/exclusion criteria unclear, probe placement not standardised, PHP severity not measured, no blinding of assessors, limited information regarding statistical analysis methods. |

*CD, cannot determine; NA, not applicable; NR, not reported

NIH Quality Assessment Tool for observational cohort and cross-sectional

| Study: **Karabay et al (2007)** |
| --- |

| Criteria | Yes | No | Other (CD, NR, NA)* |
| --- | --- | --- | --- |
| 1. Was the research question or objective in this paper clearly stated? | * |  |  |
| 2. Was the study population clearly specified and defined? | * |  |  |
| 3. Was the participation rate of eligible persons at least 50%? |  |  | NR |
| 4. Were all the subjects selected or recruited from the same or similar populations (including the same time period)? Were inclusion and exclusion criteria for being in the study prespecified and applied uniformly to all participants? |  | * | Source and selection of participants unclear  No recruitment time period mentioned  Inclusion/exclusion criteria unclear |
| 5. Was a sample size justification, power description, or variance and effect estimates provided? |  | * |  |
| 6. For the analyses in this paper, were the exposure(s) of interest measured prior to the outcome(s) being measured? |  | * | Cross-sectional study |
| 7. Was the timeframe sufficient so that one could reasonably expect to see an association between exposure and outcome if it existed? |  | * | Cross-sectional study |
| 8. For exposures that can vary in amount or level, did the study examine different levels of the exposure as related to the outcome (e.g., categories of exposure, or exposure measured as continuous variable)? | * |  |  |
| 9. Were the exposure measures (independent variables) clearly defined, valid, reliable, and implemented consistently across all study participants? |  | * | Probe placement not standardised |
| 10. Was the exposure(s) assessed more than once over time? |  | * | Cross-sectional study |
| 11. Were the outcome measures (dependent variables) clearly defined, valid, reliable, and implemented consistently across all study participants? |  | * | PHP severity not measured |
| 12. Were the outcome assessors blinded to the exposure status of participants? |  |  | NR |
| 13. Was loss to follow-up after baseline 20% or less? |  | * | Cross-sectional study |
| 14. Were key potential confounding variables measured and adjusted statistically for their impact on the relationship between exposure(s) and outcome(s) |  | * |  |
|  |  |  |  |

| **Quality Rating (Good, Fair, or Poor)** |
| --- |
| Rater #1 initials: CD: Poor |
| Rater #2 initials: MRK: Poor |
| Additional Comments (If POOR, please state why):  Source and selection of participants unclear, inclusion/exclusion criteria unclear, probe placement not standardised, PHP severity not measured, no blinding of assessors, limited information regarding statistical analysis methods and results, no adjustment for potential confounding variables. |

*CD, cannot determine; NA, not applicable; NR, not reported

NIH Quality Assessment Tool for observational cohort and cross-sectional

| Study: **Lee et al (2014)** |
| --- |

| Criteria | Yes | No | Other (CD, NR, NA)* |
| --- | --- | --- | --- |
| 1. Was the research question or objective in this paper clearly stated? | * |  |  |
| 2. Was the study population clearly specified and defined? | * |  |  |
| 3. Was the participation rate of eligible persons at least 50%? |  |  | NR |
| 4. Were all the subjects selected or recruited from the same or similar populations (including the same time period)? Were inclusion and exclusion criteria for being in the study prespecified and applied uniformly to all participants? |  | * | Source and selection of participant records unclear |
| 5. Was a sample size justification, power description, or variance and effect estimates provided? |  | * |  |
| 6. For the analyses in this paper, were the exposure(s) of interest measured prior to the outcome(s) being measured? |  | * | Retrospective study |
| 7. Was the timeframe sufficient so that one could reasonably expect to see an association between exposure and outcome if it existed? |  | * | Retrospective study |
| 8. For exposures that can vary in amount or level, did the study examine different levels of the exposure as related to the outcome (e.g., categories of exposure, or exposure measured as continuous variable)? | * |  |  |
| 9. Were the exposure measures (independent variables) clearly defined, valid, reliable, and implemented consistently across all study participants? |  | * | Probe placement not standardised |
| 10. Was the exposure(s) assessed more than once over time? |  | * | Retrospective study |
| 11. Were the outcome measures (dependent variables) clearly defined, valid, reliable, and implemented consistently across all study participants? |  | * | PHP severity not measured |
| 12. Were the outcome assessors blinded to the exposure status of participants? |  | * |  |
| 13. Was loss to follow-up after baseline 20% or less? |  | * | Retrospective study |
| 14. Were key potential confounding variables measured and adjusted statistically for their impact on the relationship between exposure(s) and outcome(s) |  | * |  |
|  |  |  |  |

| **Quality Rating (Good, Fair, or Poor)** |
| --- |
| Rater #1 initials: CD: Poor |
| Rater #2 initials: MRK: Poor |
| Additional Comments (If POOR, please state why):  Source and selection of participant records unclear, probe placement not standardised, PHP severity not measured, no blinding of assessors, no adjustment for potential confounding variables. |

*CD, cannot determine; NA, not applicable; NR, not reported

NIH Quality Assessment Tool for observational cohort and cross-sectional

| Study: **Lin et al (2015)** |
| --- |

| Criteria | Yes | No | Other (CD, NR, NA)* |
| --- | --- | --- | --- |
| 1. Was the research question or objective in this paper clearly stated? | * |  |  |
| 2. Was the study population clearly specified and defined? | * |  |  |
| 3. Was the participation rate of eligible persons at least 50%? |  |  | NR |
| 4. Were all the subjects selected or recruited from the same or similar populations (including the same time period)? Were inclusion and exclusion criteria for being in the study prespecified and applied uniformly to all participants? | * |  |  |
| 5. Was a sample size justification, power description, or variance and effect estimates provided? |  | * |  |
| 6. For the analyses in this paper, were the exposure(s) of interest measured prior to the outcome(s) being measured? |  | * | Cross-sectional study |
| 7. Was the timeframe sufficient so that one could reasonably expect to see an association between exposure and outcome if it existed? |  | * | Cross-sectional study |
| 8. For exposures that can vary in amount or level, did the study examine different levels of the exposure as related to the outcome (e.g., categories of exposure, or exposure measured as continuous variable)? | * |  |  |
| 9. Were the exposure measures (independent variables) clearly defined, valid, reliable, and implemented consistently across all study participants? | * |  |  |
| 10. Was the exposure(s) assessed more than once over time? |  | * | Cross-sectional study |
| 11. Were the outcome measures (dependent variables) clearly defined, valid, reliable, and implemented consistently across all study participants? |  | * | PHP severity not measured |
| 12. Were the outcome assessors blinded to the exposure status of participants? |  |  | NR |
| 13. Was loss to follow-up after baseline 20% or less? |  | * | Cross-sectional study |
| 14. Were key potential confounding variables measured and adjusted statistically for their impact on the relationship between exposure(s) and outcome(s) |  | * |  |
|  |  |  |  |

| **Quality Rating (Good, Fair, or Poor)** |
| --- |
| Rater #1 initials: CD: Poor |
| Rater #2 initials: MRK: Poor |
| Additional Comments (If POOR, please state why):  PHP severity not measured, no blinding of assessors, no adjustment for potential confounding variables. |

*CD, cannot determine; NA, not applicable; NR, not reported

NIH Quality Assessment Tool for observational cohort and cross-sectional

| Study: **McMillan et al (2013)** |
| --- |
|  |

| Criteria | Yes | No | Other (CD, NR, NA)* |
| --- | --- | --- | --- |
| 1. Was the research question or objective in this paper clearly stated? | * |  |  |
| 2. Was the study population clearly specified and defined? | * |  |  |
| 3. Was the participation rate of eligible persons at least 50%? |  |  | NR |
| 4. Were all the subjects selected or recruited from the same or similar populations (including the same time period)? Were inclusion and exclusion criteria for being in the study prespecified and applied uniformly to all participants? | * |  |  |
| 5. Was a sample size justification, power description, or variance and effect estimates provided? |  | * |  |
| 6. For the analyses in this paper, were the exposure(s) of interest measured prior to the outcome(s) being measured? |  | * | Cross sectional study |
| 7. Was the timeframe sufficient so that one could reasonably expect to see an association between exposure and outcome if it existed? |  | * | Cross-sectional study |
| 8. For exposures that can vary in amount or level, did the study examine different levels of the exposure as related to the outcome (e.g., categories of exposure, or exposure measured as continuous variable)? | * |  |  |
| 9. Were the exposure measures (independent variables) clearly defined, valid, reliable, and implemented consistently across all study participants? | * |  |  |
| 10. Was the exposure(s) assessed more than once over time? |  | * | Cross-sectional study |
| 11. Were the outcome measures (dependent variables) clearly defined, valid, reliable, and implemented consistently across all study participants? | * |  |  |
| 12. Were the outcome assessors blinded to the exposure status of participants? |  | * | PDU signal assessment performed by 2 blinded podiatrists  PDU image assessors not blinded |
| 13. Was loss to follow-up after baseline 20% or less? |  | * | Cross-sectional study |
| 14. Were key potential confounding variables measured and adjusted statistically for their impact on the relationship between exposure(s) and outcome(s) | * |  |  |
|  |  |  |  |

| **Quality Rating (Good, Fair, or Poor)** |
| --- |
| Rater #1 initials: Good |
| Rater #2 initials: MK: Good |
| Additional Comments (If POOR, please state why): |

*CD, cannot determine; NA, not applicable; NR, not reported

NIH Quality Assessment Tool for observational cohort and cross-sectional

Study: **Osborne et al (2006)**

| Criteria | Yes | No | Other (CD, NR, NA)* |
| --- | --- | --- | --- |
| 1. Was the research question or objective in this paper clearly stated? | * |  |  |
| 2. Was the study population clearly specified and defined? | * |  |  |
| 3. Was the participation rate of eligible persons at least 50%? |  |  | NR |
| 4. Were all the subjects selected or recruited from the same or similar populations (including the same time period)? Were inclusion and exclusion criteria for being in the study prespecified and applied uniformly to all participants? | * |  | Recruitment period not mentioned. |
| 5. Was a sample size justification, power description, or variance and effect estimates provided? |  | * |  |
| 6. For the analyses in this paper, were the exposure(s) of interest measured prior to the outcome(s) being measured? |  | * | Cross-sectional study |
| 7. Was the timeframe sufficient so that one could reasonably expect to see an association between exposure and outcome if it existed? |  | * | Cross-sectional study |
| 8. For exposures that can vary in amount or level, did the study examine different levels of the exposure as related to the outcome (e.g., categories of exposure, or exposure measured as continuous variable)? | * |  |  |
| 9. Were the exposure measures (independent variables) clearly defined, valid, reliable, and implemented consistently across all study participants? | * |  |  |
| 10. Was the exposure(s) assessed more than once over time? |  | * |  |
| 11. Were the outcome measures (dependent variables) clearly defined, valid, reliable, and implemented consistently across all study participants? |  | * | PHP severity not measured |
| 12. Were the outcome assessors blinded to the exposure status of participants? | * |  |  |
| 13. Was loss to follow-up after baseline 20% or less? |  | * | Cross-sectional study |
| 14. Were key potential confounding variables measured and adjusted statistically for their impact on the relationship between exposure(s) and outcome(s) |  | * | Age and gender measured and analysed but not BMI |
|  |  |  |  |

| **Quality Rating (Good, Fair, or Poor)** |
| --- |
| Rater #1 initials: CD: Fair |
| Rater #2 initials: MRK: Fair |
| Additional Comments (If POOR, please state why): |

*CD, cannot determine; NA, not applicable; NR, not reported

NIH Quality Assessment Tool for observational cohort and cross-sectional

| Study: **Ozdemir et al (2005)** |
| --- |

| Criteria | Yes | No | Other (CD, NR, NA)* |
| --- | --- | --- | --- |
| 1. Was the research question or objective in this paper clearly stated? | * |  |  |
| 2. Was the study population clearly specified and defined? | * |  |  |
| 3. Was the participation rate of eligible persons at least 50%? |  |  | NR |
| 4. Were all the subjects selected or recruited from the same or similar populations (including the same time period)? Were inclusion and exclusion criteria for being in the study prespecified and applied uniformly to all participants? | * |  |  |
| 5. Was a sample size justification, power description, or variance and effect estimates provided? |  | * |  |
| 6. For the analyses in this paper, were the exposure(s) of interest measured prior to the outcome(s) being measured? |  | * | Cross-sectional study |
| 7. Was the timeframe sufficient so that one could reasonably expect to see an association between exposure and outcome if it existed? |  | * | Cross-sectional study |
| 8. For exposures that can vary in amount or level, did the study examine different levels of the exposure as related to the outcome (e.g., categories of exposure, or exposure measured as continuous variable)? | * |  |  |
| 9. Were the exposure measures (independent variables) clearly defined, valid, reliable, and implemented consistently across all study participants? | * |  |  |
| 10. Was the exposure(s) assessed more than once over time? |  | * | Cross-sectional study |
| 11. Were the outcome measures (dependent variables) clearly defined, valid, reliable, and implemented consistently across all study participants? |  | * | PHP severity not measured |
| 12. Were the outcome assessors blinded to the exposure status of participants? | * |  |  |
| 13. Was loss to follow-up after baseline 20% or less? |  | * | Cross-sectional study |
| 14. Were key potential confounding variables measured and adjusted statistically for their impact on the relationship between exposure(s) and outcome(s) | * |  |  |
|  |  |  |  |

| **Quality Rating (Good, Fair, or Poor)** |
| --- |
| Rater #1 initials: CD: Good |
| Rater #2 initials: MRK: Good |
| Additional Comments (If POOR, please state why): |

*CD, cannot determine; NA, not applicable; NR, not reported

NIH Quality Assessment Tool for observational cohort and cross-sectional

| Study: **Prichasuk et al (1994)** |
| --- |

| Criteria | Yes | No | Other (CD, NR, NA)* |
| --- | --- | --- | --- |
| 1. Was the research question or objective in this paper clearly stated? | * |  |  |
| 2. Was the study population clearly specified and defined? | * |  |  |
| 3. Was the participation rate of eligible persons at least 50%? |  |  | NR |
| 4. Were all the subjects selected or recruited from the same or similar populations (including the same time period)? Were inclusion and exclusion criteria for being in the study prespecified and applied uniformly to all participants? | * |  |  |
| 5. Was a sample size justification, power description, or variance and effect estimates provided? |  | * |  |
| 6. For the analyses in this paper, were the exposure(s) of interest measured prior to the outcome(s) being measured? |  | * | Cross-sectional study |
| 7. Was the timeframe sufficient so that one could reasonably expect to see an association between exposure and outcome if it existed? |  | * | Cross-sectional study |
| 8. For exposures that can vary in amount or level, did the study examine different levels of the exposure as related to the outcome (e.g., categories of exposure, or exposure measured as continuous variable)? | * |  |  |
| 9. Were the exposure measures (independent variables) clearly defined, valid, reliable, and implemented consistently across all study participants? | * |  |  |
| 10. Was the exposure(s) assessed more than once over time? |  | * | Cross-sectional study |
| 11. Were the outcome measures (dependent variables) clearly defined, valid, reliable, and implemented consistently across all study participants? |  | * | PHP severity not measured |
| 12. Were the outcome assessors blinded to the exposure status of participants? |  | * |  |
| 13. Was loss to follow-up after baseline 20% or less? |  | * | Cross-sectional study |
| 14. Were key potential confounding variables measured and adjusted statistically for their impact on the relationship between exposure(s) and outcome(s) |  | * |  |
|  |  |  |  |

| **Quality Rating (Good, Fair, or Poor)** |
| --- |
| Rater #1 initials: CD: Poor |
| Rater #2 initials: MRK: Poor |
| Additional Comments (If POOR, please state why):  PHP severity not measured, no blinding of assessors, limited information regarding statistical analysis methods and results, no adjustment for potential confounding variables. |

*CD, cannot determine; NA, not applicable; NR, not reported

NIH Quality Assessment Tool for observational cohort and cross-sectional

| Study: **Rios-Diaz et al (2015)** |
| --- |

| Criteria | Yes | No | Other (CD, NR, NA)* |
| --- | --- | --- | --- |
| 1. Was the research question or objective in this paper clearly stated? | * |  |  |
| 2. Was the study population clearly specified and defined? | * |  |  |
| 3. Was the participation rate of eligible persons at least 50%? |  |  | NR |
| 4. Were all the subjects selected or recruited from the same or similar populations (including the same time period)? Were inclusion and exclusion criteria for being in the study prespecified and applied uniformly to all participants? | * |  |  |
| 5. Was a sample size justification, power description, or variance and effect estimates provided? | * |  |  |
| 6. For the analyses in this paper, were the exposure(s) of interest measured prior to the outcome(s) being measured? |  | * | Cross sectional study |
| 7. Was the timeframe sufficient so that one could reasonably expect to see an association between exposure and outcome if it existed? |  | * | Cross-sectional study |
| 8. For exposures that can vary in amount or level, did the study examine different levels of the exposure as related to the outcome (e.g., categories of exposure, or exposure measured as continuous variable)? | * |  |  |
| 9. Were the exposure measures (independent variables) clearly defined, valid, reliable, and implemented consistently across all study participants? | * |  |  |
| 10. Was the exposure(s) assessed more than once over time? |  | * | Cross-sectional study |
| 11. Were the outcome measures (dependent variables) clearly defined, valid, reliable, and implemented consistently across all study participants? | * |  |  |
| 12. Were the outcome assessors blinded to the exposure status of participants? | * |  |  |
| 13. Was loss to follow-up after baseline 20% or less? |  | * | Cross-sectional study |
| 14. Were key potential confounding variables measured and adjusted statistically for their impact on the relationship between exposure(s) and outcome(s) | * |  |  |
|  |  |  |  |

| **Quality Rating (Good, Fair, or Poor)** |
| --- |
| Rater #1 initials: CD: Good |
| Rater #2 initials: MK: Good |
| Additional Comments (If POOR, please state why): |

*CD, cannot determine; NA, not applicable; NR, not reported

NIH Quality Assessment Tool for Observational Cohort and Cross-Sectional Studies

| Study: **Rome et al (2002)** |
| --- |

| Criteria | Yes | No | Other (CD, NR, NA)* |
| --- | --- | --- | --- |
| 1. Was the research question or objective in this paper clearly stated? | * |  |  |
| 2. Was the study population clearly specified and defined? | * |  |  |
| 3. Was the participation rate of eligible persons at least 50%? |  |  | NR |
| 4. Were all the subjects selected or recruited from the same or similar populations (including the same time period)? Were inclusion and exclusion criteria for being in the study prespecified and applied uniformly to all participants? | * |  |  |
| 5. Was a sample size justification, power description, or variance and effect estimates provided? | * |  |  |
| 6. For the analyses in this paper, were the exposure(s) of interest measured prior to the outcome(s) being measured? |  | * | Cross sectional study |
| 7. Was the timeframe sufficient so that one could reasonably expect to see an association between exposure and outcome if it existed? |  | * | Cross-sectional study |
| 8. For exposures that can vary in amount or level, did the study examine different levels of the exposure as related to the outcome (e.g., categories of exposure, or exposure measured as continuous variable)? | * |  |  |
| 9. Were the exposure measures (independent variables) clearly defined, valid, reliable, and implemented consistently across all study participants? | * |  |  |
| 10. Was the exposure(s) assessed more than once over time? |  | * | Cross-sectional study |
| 11. Were the outcome measures (dependent variables) clearly defined, valid, reliable, and implemented consistently across all study participants? |  | * | PHP severity not measured |
| 12. Were the outcome assessors blinded to the exposure status of participants? | * |  |  |
| 13. Was loss to follow-up after baseline 20% or less? |  | * | Cross-sectional study |
| 14. Were key potential confounding variables measured and adjusted statistically for their impact on the relationship between exposure(s) and outcome(s) | * |  |  |
|  |  |  |  |

| **Quality Rating (Good, Fair, or Poor)** |
| --- |
| Rater #1 initials: CD: Good |
| Rater #2 initials: MK: Good |
| Additional Comments (If POOR, please state why): |

*CD, cannot determine; NA, not applicable; NR, not reported

NIH Quality Assessment Tool for observational cohort and cross-sectional

| Study: **Sabir et al (2005)** |
| --- |

| Criteria | Yes | No | Other (CD, NR, NA)* |
| --- | --- | --- | --- |
| 1. Was the research question or objective in this paper clearly stated? | * |  |  |
| 2. Was the study population clearly specified and defined? | * |  |  |
| 3. Was the participation rate of eligible persons at least 50%? |  |  | NR |
| 4. Were all the subjects selected or recruited from the same or similar populations (including the same time period)? Were inclusion and exclusion criteria for being in the study prespecified and applied uniformly to all participants? |  | * | Source and selection of participants unclear  No recruitment time period mentioned  Inclusion/exclusion criteria unclear |
| 5. Was a sample size justification, power description, or variance and effect estimates provided? |  | * |  |
| 6. For the analyses in this paper, were the exposure(s) of interest measured prior to the outcome(s) being measured? |  | * | Cross-sectional study |
| 7. Was the timeframe sufficient so that one could reasonably expect to see an association between exposure and outcome if it existed? |  | * | Cross-sectional study |
| 8. For exposures that can vary in amount or level, did the study examine different levels of the exposure as related to the outcome (e.g., categories of exposure, or exposure measured as continuous variable)? | * |  |  |
| 9. Were the exposure measures (independent variables) clearly defined, valid, reliable, and implemented consistently across all study participants? | * |  |  |
| 10. Was the exposure(s) assessed more than once over time? |  | * | Cross-sectional study |
| 11. Were the outcome measures (dependent variables) clearly defined, valid, reliable, and implemented consistently across all study participants? |  | * | PHP severity not measured |
| 12. Were the outcome assessors blinded to the exposure status of participants? | * |  |  |
| 13. Was loss to follow-up after baseline 20% or less? |  | * | Cross-sectional study |
| 14. Were key potential confounding variables measured and adjusted statistically for their impact on the relationship between exposure(s) and outcome(s) | * |  | Matched controls (age, sex) |
|  |  |  |  |

| **Quality Rating (Good, Fair, or Poor)** |
| --- |
| Rater #1 initials: CD: Fair |
| Rater #2 initials: MRK: Fair |
| Additional Comments (If POOR, please state why): |

*CD, cannot determine; NA, not applicable; NR, not reported

NIH Quality Assessment Tool for observational cohort and cross-sectional

| Study: **Sahin et al (2010)** |
| --- |

| Criteria | Yes | No | Other (CD, NR, NA)* |
| --- | --- | --- | --- |
| 1. Was the research question or objective in this paper clearly stated? | * |  |  |
| 2. Was the study population clearly specified and defined? | * |  |  |
| 3. Was the participation rate of eligible persons at least 50%? |  |  | NR |
| 4. Were all the subjects selected or recruited from the same or similar populations (including the same time period)? Were inclusion and exclusion criteria for being in the study prespecified and applied uniformly to all participants? | * |  |  |
| 5. Was a sample size justification, power description, or variance and effect estimates provided? |  | * |  |
| 6. For the analyses in this paper, were the exposure(s) of interest measured prior to the outcome(s) being measured? |  | * | Cross-sectional study |
| 7. Was the timeframe sufficient so that one could reasonably expect to see an association between exposure and outcome if it existed? |  | * | Cross-sectional study |
| 8. For exposures that can vary in amount or level, did the study examine different levels of the exposure as related to the outcome (e.g., categories of exposure, or exposure measured as continuous variable)? | * |  |  |
| 9. Were the exposure measures (independent variables) clearly defined, valid, reliable, and implemented consistently across all study participants? | * |  |  |
| 10. Was the exposure(s) assessed more than once over time? |  | * | Cross-sectional study |
| 11. Were the outcome measures (dependent variables) clearly defined, valid, reliable, and implemented consistently across all study participants? |  | * | PHP severity not measured |
| 12. Were the outcome assessors blinded to the exposure status of participants? |  |  | NR |
| 13. Was loss to follow-up after baseline 20% or less? |  | * | Cross-sectional study |
| 14. Were key potential confounding variables measured and adjusted statistically for their impact on the relationship between exposure(s) and outcome(s) |  | * |  |
|  |  |  |  |

| **Quality Rating (Good, Fair, or Poor)** |
| --- |
| Rater #1 initials: CD: Poor |
| Rater #2 initials: MRK: Poor |
| Additional Comments (If POOR, please state why):  PHP severity not measured, no blinding of assessors, no adjustment for potential confounding variables |

*CD, cannot determine; NA, not applicable; NR, not reported

NIH Quality Assessment Tool for observational cohort and cross-sectional

| Study: **Schillizzi et al (2020)** |
| --- |

| Criteria | Yes | No | Other (CD, NR, NA)* |
| --- | --- | --- | --- |
| 1. Was the research question or objective in this paper clearly stated? | * |  |  |
| 2. Was the study population clearly specified and defined? | * |  |  |
| 3. Was the participation rate of eligible persons at least 50%? |  |  | NR |
| 4. Were all the subjects selected or recruited from the same or similar populations (including the same time period)? Were inclusion and exclusion criteria for being in the study prespecified and applied uniformly to all participants? | * |  |  |
| 5. Was a sample size justification, power description, or variance and effect estimates provided? |  | * |  |
| 6. For the analyses in this paper, were the exposure(s) of interest measured prior to the outcome(s) being measured? |  | * | Cross-sectional study |
| 7. Was the timeframe sufficient so that one could reasonably expect to see an association between exposure and outcome if it existed? |  | * | Cross-sectional study |
| 8. For exposures that can vary in amount or level, did the study examine different levels of the exposure as related to the outcome (e.g., categories of exposure, or exposure measured as continuous variable)? | * |  |  |
| 9. Were the exposure measures (independent variables) clearly defined, valid, reliable, and implemented consistently across all study participants? | * |  |  |
| 10. Was the exposure(s) assessed more than once over time? |  | * | Cross-sectional study |
| 11. Were the outcome measures (dependent variables) clearly defined, valid, reliable, and implemented consistently across all study participants? | * |  |  |
| 12. Were the outcome assessors blinded to the exposure status of participants? |  |  | NR |
| 13. Was loss to follow-up after baseline 20% or less? |  | * | Cross-sectional study |
| 14. Were key potential confounding variables measured and adjusted statistically for their impact on the relationship between exposure(s) and outcome(s) | * |  | Similar participant characteristics |
|  |  |  |  |

| **Quality Rating (Good, Fair, or Poor)** |
| --- |
| Rater #1 initials: CD: Fair |
| Rater #2 initials: MRK: Fair |
| Additional Comments (If POOR, please state why): |

*CD, cannot determine; NA, not applicable; NR, not reported

NIH Quality Assessment Tool for observational cohort and cross-sectional

| Study: **Sconfienza et al (2013)** |
| --- |

| Criteria | Yes | No | Other (CD, NR, NA)* |
| --- | --- | --- | --- |
| 1. Was the research question or objective in this paper clearly stated? | * |  |  |
| 2. Was the study population clearly specified and defined? | * |  |  |
| 3. Was the participation rate of eligible persons at least 50%? |  |  | NR |
| 4. Were all the subjects selected or recruited from the same or similar populations (including the same time period)? Were inclusion and exclusion criteria for being in the study prespecified and applied uniformly to all participants? | * |  |  |
| 5. Was a sample size justification, power description, or variance and effect estimates provided? |  | * |  |
| 6. For the analyses in this paper, were the exposure(s) of interest measured prior to the outcome(s) being measured? |  | * | Cross-sectional study |
| 7. Was the timeframe sufficient so that one could reasonably expect to see an association between exposure and outcome if it existed? |  | * | Cross-sectional study |
| 8. For exposures that can vary in amount or level, did the study examine different levels of the exposure as related to the outcome (e.g., categories of exposure, or exposure measured as continuous variable)? | * |  |  |
| 9. Were the exposure measures (independent variables) clearly defined, valid, reliable, and implemented consistently across all study participants? | * |  |  |
| 10. Was the exposure(s) assessed more than once over time? |  | * | Cross-sectional study |
| 11. Were the outcome measures (dependent variables) clearly defined, valid, reliable, and implemented consistently across all study participants? | * |  |  |
| 12. Were the outcome assessors blinded to the exposure status of participants? | * |  |  |
| 13. Was loss to follow-up after baseline 20% or less? |  | * | Cross-sectional study |
| 14. Were key potential confounding variables measured and adjusted statistically for their impact on the relationship between exposure(s) and outcome(s) | * |  | Similar participant characteristics |
|  |  |  |  |

| **Quality Rating (Good, Fair, or Poor)** |
| --- |
| Rater #1 initials: CD: Good |
| Rater #2 initials: MRK: Good |
| Additional Comments (If POOR, please state why): |

*CD, cannot determine; NA, not applicable; NR, not reported

NIH Quality Assessment Tool for observational cohort and cross-sectional

| Study: **Song et al (2019)** |
| --- |

| Criteria | Yes | No | Other (CD, NR, NA)* |
| --- | --- | --- | --- |
| 1. Was the research question or objective in this paper clearly stated? | * |  |  |
| 2. Was the study population clearly specified and defined? | * |  |  |
| 3. Was the participation rate of eligible persons at least 50%? |  |  | NR |
| 4. Were all the subjects selected or recruited from the same or similar populations (including the same time period)? Were inclusion and exclusion criteria for being in the study prespecified and applied uniformly to all participants? | * |  |  |
| 5. Was a sample size justification, power description, or variance and effect estimates provided? |  | * |  |
| 6. For the analyses in this paper, were the exposure(s) of interest measured prior to the outcome(s) being measured? |  | * | Cross-sectional study |
| 7. Was the timeframe sufficient so that one could reasonably expect to see an association between exposure and outcome if it existed? |  | * | Cross-sectional study |
| 8. For exposures that can vary in amount or level, did the study examine different levels of the exposure as related to the outcome (e.g., categories of exposure, or exposure measured as continuous variable)? | * |  |  |
| 9. Were the exposure measures (independent variables) clearly defined, valid, reliable, and implemented consistently across all study participants? | * |  |  |
| 10. Was the exposure(s) assessed more than once over time? |  | * | Cross-sectional study |
| 11. Were the outcome measures (dependent variables) clearly defined, valid, reliable, and implemented consistently across all study participants? |  | * | PHP severity not measured |
| 12. Were the outcome assessors blinded to the exposure status of participants? |  |  | NR |
| 13. Was loss to follow-up after baseline 20% or less? |  | * | Cross-sectional study |
| 14. Were key potential confounding variables measured and adjusted statistically for their impact on the relationship between exposure(s) and outcome(s) | * |  | Similar participant characteristics |
|  |  |  |  |

| **Quality Rating (Good, Fair, or Poor)** |
| --- |
| Rater #1 initials: CD: Fair |
| Rater #2 initials: MRK: Fair |
| Additional Comments (If POOR, please state why): |

*CD, cannot determine; NA, not applicable; NR, not reported

NIH Quality Assessment Tool for observational cohort and cross-sectional

| Study: **Sutera et al (2010)** |
| --- |

| Criteria | Yes | No | Other (CD, NR, NA)* |
| --- | --- | --- | --- |
| 1. Was the research question or objective in this paper clearly stated? | * |  |  |
| 2. Was the study population clearly specified and defined? | * |  |  |
| 3. Was the participation rate of eligible persons at least 50%? |  |  | NR |
| 4. Were all the subjects selected or recruited from the same or similar populations (including the same time period)? Were inclusion and exclusion criteria for being in the study prespecified and applied uniformly to all participants? |  | * | Source and selection of participants unclear |
| 5. Was a sample size justification, power description, or variance and effect estimates provided? |  | * |  |
| 6. For the analyses in this paper, were the exposure(s) of interest measured prior to the outcome(s) being measured? |  | * | Cross-sectional study |
| 7. Was the timeframe sufficient so that one could reasonably expect to see an association between exposure and outcome if it existed? |  | * | Cross-sectional study |
| 8. For exposures that can vary in amount or level, did the study examine different levels of the exposure as related to the outcome (e.g., categories of exposure, or exposure measured as continuous variable)? | * |  |  |
| 9. Were the exposure measures (independent variables) clearly defined, valid, reliable, and implemented consistently across all study participants? | * |  |  |
| 10. Was the exposure(s) assessed more than once over time? |  | * | Cross-sectional study |
| 11. Were the outcome measures (dependent variables) clearly defined, valid, reliable, and implemented consistently across all study participants? |  | * | PHP severity not measured |
| 12. Were the outcome assessors blinded to the exposure status of participants? | * |  |  |
| 13. Was loss to follow-up after baseline 20% or less? |  | * | Cross-sectional study |
| 14. Were key potential confounding variables measured and adjusted statistically for their impact on the relationship between exposure(s) and outcome(s) |  | * |  |
|  |  |  |  |

| **Quality Rating (Good, Fair, or Poor)** |
| --- |
| Rater #1 initials: CD: Fair |
| Rater #2 initials: MRK: Fair |
| Additional Comments (If POOR, please state why):  Source and selection of participants unclear, PHP severity not measured, no adjustment for potential confounding variables. |

*CD, cannot determine; NA, not applicable; NR, not reported

NIH Quality Assessment Tool for observational cohort and cross-sectional

| Study: **Tsai et al (2000)** |
| --- |

| Criteria | Yes | No | Other (CD, NR, NA)* |
| --- | --- | --- | --- |
| 1. Was the research question or objective in this paper clearly stated? | * |  |  |
| 2. Was the study population clearly specified and defined? | * |  |  |
| 3. Was the participation rate of eligible persons at least 50%? |  |  | NR |
| 4. Were all the subjects selected or recruited from the same or similar populations (including the same time period)? Were inclusion and exclusion criteria for being in the study prespecified and applied uniformly to all participants? |  | * | Source and selection of participants unclear  No recruitment time period mentioned |
| 5. Was a sample size justification, power description, or variance and effect estimates provided? |  | * |  |
| 6. For the analyses in this paper, were the exposure(s) of interest measured prior to the outcome(s) being measured? |  | * | Cross-sectional study |
| 7. Was the timeframe sufficient so that one could reasonably expect to see an association between exposure and outcome if it existed? |  | * | Cross-sectional study |
| 8. For exposures that can vary in amount or level, did the study examine different levels of the exposure as related to the outcome (e.g., categories of exposure, or exposure measured as continuous variable)? | * |  |  |
| 9. Were the exposure measures (independent variables) clearly defined, valid, reliable, and implemented consistently across all study participants? | * |  |  |
| 10. Was the exposure(s) assessed more than once over time? |  | * | Cross-sectional study |
| 11. Were the outcome measures (dependent variables) clearly defined, valid, reliable, and implemented consistently across all study participants? |  | * | PHP severity not measured |
| 12. Were the outcome assessors blinded to the exposure status of participants? | * |  |  |
| 13. Was loss to follow-up after baseline 20% or less? |  | * | Cross-sectional study |
| 14. Were key potential confounding variables measured and adjusted statistically for their impact on the relationship between exposure(s) and outcome(s) | * |  |  |
|  |  |  |  |

| **Quality Rating (Good, Fair, or Poor)** |
| --- |
| Rater #1 initials: CD: Fair |
| Rater #2 initials: MRK: Fair |
| Additional Comments (If POOR, please state why): |

*CD, cannot determine; NA, not applicable; NR, not reported

NIH Quality Assessment Tool for observational cohort and cross-sectional

| Study: **Turgut et al (1999)** |
| --- |

| Criteria | Yes | No | Other (CD, NR, NA)* |
| --- | --- | --- | --- |
| 1. Was the research question or objective in this paper clearly stated? | * |  |  |
| 2. Was the study population clearly specified and defined? | * |  |  |
| 3. Was the participation rate of eligible persons at least 50%? |  |  | NR |
| 4. Were all the subjects selected or recruited from the same or similar populations (including the same time period)? Were inclusion and exclusion criteria for being in the study prespecified and applied uniformly to all participants? | * |  |  |
| 5. Was a sample size justification, power description, or variance and effect estimates provided? |  | * |  |
| 6. For the analyses in this paper, were the exposure(s) of interest measured prior to the outcome(s) being measured? |  | * | Cross-sectional study |
| 7. Was the timeframe sufficient so that one could reasonably expect to see an association between exposure and outcome if it existed? |  | * | Cross-sectional study |
| 8. For exposures that can vary in amount or level, did the study examine different levels of the exposure as related to the outcome (e.g., categories of exposure, or exposure measured as continuous variable)? | * |  |  |
| 9. Were the exposure measures (independent variables) clearly defined, valid, reliable, and implemented consistently across all study participants? | * |  |  |
| 10. Was the exposure(s) assessed more than once over time? |  | * | Cross-sectional study |
| 11. Were the outcome measures (dependent variables) clearly defined, valid, reliable, and implemented consistently across all study participants? |  | * | PHP severity not measured |
| 12. Were the outcome assessors blinded to the exposure status of participants? | * |  |  |
| 13. Was loss to follow-up after baseline 20% or less? |  | * | Cross-sectional study |
| 14. Were key potential confounding variables measured and adjusted statistically for their impact on the relationship between exposure(s) and outcome(s) | * |  |  |
|  |  |  |  |

| **Quality Rating (Good, Fair, or Poor)** |
| --- |
| Rater #1 initials: Good |
| Rater #2 initials: MRK: Good |
| Additional Comments (If POOR, please state why): |

*CD, cannot determine; NA, not applicable; NR, not reported

NIH Quality Assessment Tool for observational cohort and cross-sectional

| Study: **Wall et al (1993)** |
| --- |

| Criteria | Yes | No | Other (CD, NR, NA)* |
| --- | --- | --- | --- |
| 1. Was the research question or objective in this paper clearly stated? | * |  |  |
| 2. Was the study population clearly specified and defined? | * |  |  |
| 3. Was the participation rate of eligible persons at least 50%? |  |  | NR |
| 4. Were all the subjects selected or recruited from the same or similar populations (including the same time period)? Were inclusion and exclusion criteria for being in the study prespecified and applied uniformly to all participants? | * |  |  |
| 5. Was a sample size justification, power description, or variance and effect estimates provided? |  | * |  |
| 6. For the analyses in this paper, were the exposure(s) of interest measured prior to the outcome(s) being measured? |  | * | Cross-sectional study |
| 7. Was the timeframe sufficient so that one could reasonably expect to see an association between exposure and outcome if it existed? |  | * | Cross-sectional study |
| 8. For exposures that can vary in amount or level, did the study examine different levels of the exposure as related to the outcome (e.g., categories of exposure, or exposure measured as continuous variable)? | * |  |  |
| 9. Were the exposure measures (independent variables) clearly defined, valid, reliable, and implemented consistently across all study participants? | * |  |  |
| 10. Was the exposure(s) assessed more than once over time? |  | * | Cross-sectional study |
| 11. Were the outcome measures (dependent variables) clearly defined, valid, reliable, and implemented consistently across all study participants? |  | * | PHP severity not measured |
| 12. Were the outcome assessors blinded to the exposure status of participants? | * |  |  |
| 13. Was loss to follow-up after baseline 20% or less? |  | * | Cross-sectional study |
| 14. Were key potential confounding variables measured and adjusted statistically for their impact on the relationship between exposure(s) and outcome(s) |  | * |  |
|  |  |  |  |

| **Quality Rating (Good, Fair, or Poor)** |
| --- |
| Rater #1 initials: CD: Fair |
| Rater #2 initials: MRK: Fair |
| Additional Comments (If POOR, please state why): |

*CD, cannot determine; NA, not applicable; NR, not reported

NIH Quality Assessment Tool for observational cohort and cross-sectional

| Study: **Walther et al (2004)** |
| --- |

| Criteria | Yes | No | Other (CD, NR, NA)* |
| --- | --- | --- | --- |
| 1. Was the research question or objective in this paper clearly stated? | * |  |  |
| 2. Was the study population clearly specified and defined? | * |  |  |
| 3. Was the participation rate of eligible persons at least 50%? |  |  | NR |
| 4. Were all the subjects selected or recruited from the same or similar populations (including the same time period)? Were inclusion and exclusion criteria for being in the study prespecified and applied uniformly to all participants? |  | * | Source and selection of participants unclear  No recruitment time period mentioned |
| 5. Was a sample size justification, power description, or variance and effect estimates provided? |  | * |  |
| 6. For the analyses in this paper, were the exposure(s) of interest measured prior to the outcome(s) being measured? |  | * | Cross-sectional study |
| 7. Was the timeframe sufficient so that one could reasonably expect to see an association between exposure and outcome if it existed? |  | * | Cross-sectional study |
| 8. For exposures that can vary in amount or level, did the study examine different levels of the exposure as related to the outcome (e.g., categories of exposure, or exposure measured as continuous variable)? | * |  |  |
| 9. Were the exposure measures (independent variables) clearly defined, valid, reliable, and implemented consistently across all study participants? | * |  |  |
| 10. Was the exposure(s) assessed more than once over time? |  | * | Cross-sectional study |
| 11. Were the outcome measures (dependent variables) clearly defined, valid, reliable, and implemented consistently across all study participants? | * |  |  |
| 12. Were the outcome assessors blinded to the exposure status of participants? |  |  | NR |
| 13. Was loss to follow-up after baseline 20% or less? |  | * | Cross-sectional study |
| 14. Were key potential confounding variables measured and adjusted statistically for their impact on the relationship between exposure(s) and outcome(s) |  | * |  |
|  |  |  |  |

| **Quality Rating (Good, Fair, or Poor)** |
| --- |
| Rater #1 initials: CD: Poor |
| Rater #2 initials: MRK: Poor |
| Additional Comments (If POOR, please state why):  Source and selection of participants unclear, no blinding of assessors, limited information regarding statistical analysis methods and results, no adjustment for potential confounding variables. |

*CD, cannot determine; NA, not applicable; NR, not reported

NIH Quality Assessment Tool for observational cohort and cross-sectional

| Study: **Wearing et al (2007)** |
| --- |

| Criteria | Yes | No | Other (CD, NR, NA)* |
| --- | --- | --- | --- |
| 1. Was the research question or objective in this paper clearly stated? | * |  |  |
| 2. Was the study population clearly specified and defined? | * |  |  |
| 3. Was the participation rate of eligible persons at least 50%? |  |  | NR |
| 4. Were all the subjects selected or recruited from the same or similar populations (including the same time period)? Were inclusion and exclusion criteria for being in the study prespecified and applied uniformly to all participants? |  | * | Source and selection of participants unclear  No recruitment time period mentioned |
| 5. Was a sample size justification, power description, or variance and effect estimates provided? |  | * |  |
| 6. For the analyses in this paper, were the exposure(s) of interest measured prior to the outcome(s) being measured? |  | * | Cross-sectional study |
| 7. Was the timeframe sufficient so that one could reasonably expect to see an association between exposure and outcome if it existed? |  | * | Cross-sectional study |
| 8. For exposures that can vary in amount or level, did the study examine different levels of the exposure as related to the outcome (e.g., categories of exposure, or exposure measured as continuous variable)? | * |  |  |
| 9. Were the exposure measures (independent variables) clearly defined, valid, reliable, and implemented consistently across all study participants? | * |  |  |
| 10. Was the exposure(s) assessed more than once over time? |  | * | Cross-sectional study |
| 11. Were the outcome measures (dependent variables) clearly defined, valid, reliable, and implemented consistently across all study participants? | * |  |  |
| 12. Were the outcome assessors blinded to the exposure status of participants? |  |  | NR |
| 13. Was loss to follow-up after baseline 20% or less? |  | * | Cross-sectional study |
| 14. Were key potential confounding variables measured and adjusted statistically for their impact on the relationship between exposure(s) and outcome(s) | * |  | Matched controls for age, height and weight |
|  |  |  |  |

| **Quality Rating (Good, Fair, or Poor)** |
| --- |
| Rater #1 initials: CD: Fair |
| Rater #2 initials: MRK: Fair |
| Additional Comments (If POOR, please state why): |

*CD, cannot determine; NA, not applicable; NR, not reported

NIH Quality Assessment Tool for observational cohort and cross-sectional

| Study: **Wearing et al (2010)** |
| --- |

| Criteria | Yes | No | Other (CD, NR, NA)* |
| --- | --- | --- | --- |
| 1. Was the research question or objective in this paper clearly stated? | * |  |  |
| 2. Was the study population clearly specified and defined? | * |  |  |
| 3. Was the participation rate of eligible persons at least 50%? |  |  | NR |
| 4. Were all the subjects selected or recruited from the same or similar populations (including the same time period)? Were inclusion and exclusion criteria for being in the study prespecified and applied uniformly to all participants? | * |  |  |
| 5. Was a sample size justification, power description, or variance and effect estimates provided? |  | * |  |
| 6. For the analyses in this paper, were the exposure(s) of interest measured prior to the outcome(s) being measured? |  | * | Cross-sectional study |
| 7. Was the timeframe sufficient so that one could reasonably expect to see an association between exposure and outcome if it existed? |  | * | Cross-sectional study |
| 8. For exposures that can vary in amount or level, did the study examine different levels of the exposure as related to the outcome (e.g., categories of exposure, or exposure measured as continuous variable)? | * |  |  |
| 9. Were the exposure measures (independent variables) clearly defined, valid, reliable, and implemented consistently across all study participants? | * |  |  |
| 10. Was the exposure(s) assessed more than once over time? |  | * | Cross-sectional study |
| 11. Were the outcome measures (dependent variables) clearly defined, valid, reliable, and implemented consistently across all study participants? | * |  |  |
| 12. Were the outcome assessors blinded to the exposure status of participants? | * |  |  |
| 13. Was loss to follow-up after baseline 20% or less? |  | * | Cross-sectional study |
| 14. Were key potential confounding variables measured and adjusted statistically for their impact on the relationship between exposure(s) and outcome(s) | * |  | Matched controls (age, sex, weight) |
|  |  |  |  |

| **Quality Rating (Good, Fair, or Poor)** |
| --- |
| Rater #1 initials: CD: Good |
| Rater #2 initials: MRK: Good |
| Additional Comments (If POOR, please state why): |

*CD, cannot determine; NA, not applicable; NR, not reported

NIH Quality Assessment Tool for observational cohort and cross-sectional

| Study: **Williams et al (1987)** |
| --- |

| Criteria | Yes | No | Other (CD, NR, NA)* |
| --- | --- | --- | --- |
| 1. Was the research question or objective in this paper clearly stated? |  | * |  |
| 2. Was the study population clearly specified and defined? | * |  |  |
| 3. Was the participation rate of eligible persons at least 50%? |  |  | NR |
| 4. Were all the subjects selected or recruited from the same or similar populations (including the same time period)? Were inclusion and exclusion criteria for being in the study prespecified and applied uniformly to all participants? | * |  |  |
| 5. Was a sample size justification, power description, or variance and effect estimates provided? |  | * |  |
| 6. For the analyses in this paper, were the exposure(s) of interest measured prior to the outcome(s) being measured? |  | * | Cross-sectional study |
| 7. Was the timeframe sufficient so that one could reasonably expect to see an association between exposure and outcome if it existed? |  | * | Cross-sectional study |
| 8. For exposures that can vary in amount or level, did the study examine different levels of the exposure as related to the outcome (e.g., categories of exposure, or exposure measured as continuous variable)? | * |  |  |
| 9. Were the exposure measures (independent variables) clearly defined, valid, reliable, and implemented consistently across all study participants? | * |  |  |
| 10. Was the exposure(s) assessed more than once over time? |  | * | Cross-sectional study |
| 11. Were the outcome measures (dependent variables) clearly defined, valid, reliable, and implemented consistently across all study participants? |  | * | PHP severity not measured |
| 12. Were the outcome assessors blinded to the exposure status of participants? | * |  |  |
| 13. Was loss to follow-up after baseline 20% or less? |  | * | Cross-sectional study |
| 14. Were key potential confounding variables measured and adjusted statistically for their impact on the relationship between exposure(s) and outcome(s) |  | * |  |
|  |  |  |  |

| **Quality Rating (Good, Fair, or Poor)** |
| --- |
| Rater #1 initials: CD: Poor |
| Rater #2 initials: MRK: Poor |
| Additional Comments (If POOR, please state why):  Research question and objectives unclear, PHP severity not measured, limited information regarding statistical analysis methods and results, no adjustment for potential confounding variables. |

*CD, cannot determine; NA, not applicable; NR, not reported

NIH Quality Assessment Tool for observational cohort and cross-sectional

| Study: **Wu et al (2011)** |
| --- |

| Criteria | Yes | No | Other (CD, NR, NA)* |
| --- | --- | --- | --- |
| 1. Was the research question or objective in this paper clearly stated? | * |  |  |
| 2. Was the study population clearly specified and defined? | * |  |  |
| 3. Was the participation rate of eligible persons at least 50%? |  |  | NR |
| 4. Were all the subjects selected or recruited from the same or similar populations (including the same time period)? Were inclusion and exclusion criteria for being in the study prespecified and applied uniformly to all participants? | * |  |  |
| 5. Was a sample size justification, power description, or variance and effect estimates provided? |  | * |  |
| 6. For the analyses in this paper, were the exposure(s) of interest measured prior to the outcome(s) being measured? |  | * | Cross-sectional study |
| 7. Was the timeframe sufficient so that one could reasonably expect to see an association between exposure and outcome if it existed? |  | * | Cross-sectional study |
| 8. For exposures that can vary in amount or level, did the study examine different levels of the exposure as related to the outcome (e.g., categories of exposure, or exposure measured as continuous variable)? | * |  |  |
| 9. Were the exposure measures (independent variables) clearly defined, valid, reliable, and implemented consistently across all study participants? | * |  |  |
| 10. Was the exposure(s) assessed more than once over time? |  | * | Cross-sectional study |
| 11. Were the outcome measures (dependent variables) clearly defined, valid, reliable, and implemented consistently across all study participants? | * |  |  |
| 12. Were the outcome assessors blinded to the exposure status of participants? |  | * |  |
| 13. Was loss to follow-up after baseline 20% or less? |  | * | Cross-sectional study |
| 14. Were key potential confounding variables measured and adjusted statistically for their impact on the relationship between exposure(s) and outcome(s) | * |  |  |
|  |  |  |  |

| **Quality Rating (Good, Fair, or Poor)** |
| --- |
| Rater #1 initials: CD: Fair |
| Rater #2 initials: MRK: Fair |
| Additional Comments (If POOR, please state why): |

*CD, cannot determine; NA, not applicable; NR, not reported

NIH Quality Assessment Tool for observational cohort and cross-sectional

| Study: **Wu et al (2015)** |
| --- |

| Criteria | Yes | No | Other (CD, NR, NA)* |
| --- | --- | --- | --- |
| 1. Was the research question or objective in this paper clearly stated? | * |  |  |
| 2. Was the study population clearly specified and defined? | * |  |  |
| 3. Was the participation rate of eligible persons at least 50%? |  |  | NR |
| 4. Were all the subjects selected or recruited from the same or similar populations (including the same time period)? Were inclusion and exclusion criteria for being in the study prespecified and applied uniformly to all participants? | * |  |  |
| 5. Was a sample size justification, power description, or variance and effect estimates provided? |  | * |  |
| 6. For the analyses in this paper, were the exposure(s) of interest measured prior to the outcome(s) being measured? |  | * | Cross-sectional study |
| 7. Was the timeframe sufficient so that one could reasonably expect to see an association between exposure and outcome if it existed? |  | * | Cross-sectional study |
| 8. For exposures that can vary in amount or level, did the study examine different levels of the exposure as related to the outcome (e.g., categories of exposure, or exposure measured as continuous variable)? | * |  |  |
| 9. Were the exposure measures (independent variables) clearly defined, valid, reliable, and implemented consistently across all study participants? | * |  |  |
| 10. Was the exposure(s) assessed more than once over time? |  | * | Cross-sectional study |
| 11. Were the outcome measures (dependent variables) clearly defined, valid, reliable, and implemented consistently across all study participants? | * |  |  |
| 12. Were the outcome assessors blinded to the exposure status of participants? | * |  |  |
| 13. Was loss to follow-up after baseline 20% or less? |  | * | Cross-sectional study |
| 14. Were key potential confounding variables measured and adjusted statistically for their impact on the relationship between exposure(s) and outcome(s) | * |  | Similar characteristics between groups |
|  |  |  |  |

| **Quality Rating (Good, Fair, or Poor)** |
| --- |
| Rater #1 initials: CD: Good |
| Rater #2 initials: MRK: Good |
| Additional Comments (If POOR, please state why): |

*CD, cannot determine; NA, not applicable; NR, not reported
